# Adaptive radiation of the *Callicarpa* genus in the Bonin Islands revealed through double‐digest restriction site‐associated DNA sequencing analysis

**DOI:** 10.1002/ece3.70216

**Published:** 2024-09-13

**Authors:** Suzuki Setsuko, Satoshi Narita, Ichiro Tamaki, Kyoko Sugai, Atsushi J. Nagano, Tokuko Ujino‐Ihara, Hidetoshi Kato, Yuji Isagi

**Affiliations:** ^1^ Department of Forest Molecular Genetics and Biotechnology, Forestry and Forest Products Research Institute Forest Research and Management Organization Tsukuba Ibaraki Japan; ^2^ Graduate School of Agriculture Kyoto University Sakyo‐ku Kyoto Japan; ^3^ Faculty of Applied Biological Sciences, Gifu Field Science Center Gifu University Gifu Gifu Japan; ^4^ Institute of Agricultural and Life Sciences Academic Assembly, Shimane University Matsue Shimane Japan; ^5^ Faculty of Agriculture Ryukoku University Otsu Shiga Japan; ^6^ Institute for Advanced Biosciences Keio University Tsuruoka Yamagata Japan; ^7^ Makino Herbarium Tokyo Metropolitan University Hachioji Tokyo Japan

**Keywords:** ddRAD‐Seq, Lamiaceae, oceanic islands, Ogasawara Islands, quaternary

## Abstract

The Bonin Islands, comprised of the Mukojima, Chichijima, and Hahajima Islands, are known for their isolated and distinctive habitats, hosting a diverse array of endemic flora and fauna. In these islands, adaptive radiation has played a remarkable role in speciation, particularly evident in the *Callicarpa* genus that is represented by three species: *Callicarpa parvifolia* and *C. glabra* exclusive to the Chichijima Islands, and *Callicarpa subpubescens*, distributed across the entire Bonin Islands. Notably, *C. subpubescens* exhibits multiple ecotypes, differing in leaf hair density, flowering time, and tree size. In this study, we aimed to investigate species and ecotype diversification patterns, estimate divergence times, and explore cryptic species within *Callicarpa* in the Bonin Islands using phenotypic and genetic data (double‐digest restriction site‐associated DNA sequencing). Genetic analysis revealed that *C. parvifolia* and *C. glabra* both formed single, distinct genetic groups. Conversely, *C. subpubescens* consisted of six genetic groups corresponding to different ecotypes and regions, and a hybrid group resulting from the hybridization between two of these genetic groups. Population demography analysis focusing on six Chichijima and Hahajima Islands‐based species/ecotypes indicated that all species and ecotypes except one ecotype diverged simultaneously around 73–77 kya. The star‐shaped neighbor‐net tree also suggests the simultaneous divergence of species and ecotypes. The species and ecotypes that simultaneously diverged adapted to dry environments and understory forests, suggesting that aridification may have contributed to this process of adaptive radiation. Moreover, leaf morphology, flowering time, and genetic analyses suggested the presence of two cryptic species and one hybrid species within *C. subpubescens*.

## Graphical abstract

This study investigated the keystone endemic tree genus *Callicarpa* in the Bonin Islands, revealing two cryptic species and one hybrid species based on phenotypic, genotypic, and phylogenetic patterns. It provides evidence of simultaneous divergence of multiple species and ecotypes, offering definitive evidence of adaptive radiation and suggesting a link to global environmental changes.

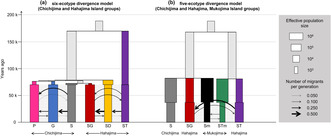

## 1 INTRODUCTION

Oceanic islands are geographically isolated—never having had a connection to large landmasses. Owing to their distinctive characteristics and limited colonization opportunities, the biotas of oceanic islands exhibit a greater degree of endemic flora and fauna, often resulting from frequent ecological speciation and adaptive radiation (Gillespie et al., 2001; Givnish, 1997). Adaptive radiation refers to the rapid diversification of lineages into multiple taxa, each adapted to distinct ecological niches (Rundle & Nosil, 2005; Schluter, 2000). Some examples include Darwin's finches in the Galapagos Islands (Grant, 1986, 1998; Grant & Grant, 2007) and silverswords and Hawaiian lobeliads in the Hawaiian Islands (Carlquist et al., 2003; Givnish et al., 2009).

The Bonin Islands, comprised of the Mukojima, Chichijima, and Hahajima Islands, are oceanic islands located approximately 1000 km south of Tokyo, Japan (Figure S1). Despite their smaller land area (70 km^2^) and lower maximum altitude (463 m) compared with other oceanic islands, such as the Canary Islands (area, 7490 km^2^; elevation, 3718 m) and the Galapagos Islands (area, 7870 km^2^; 1707 m), the Bonin Islands exhibit a high proportion of endemic vascular flora, with ~43% of species being endemic (Ono et al., 1986), exceeding 26% and 43% endemism in the Canary Islands (Aedo et al., 2013) and the Galapagos Islands (Porter, 1978), respectively. Diverse habitats, including forests, grasslands, and coastal areas, providing varied microclimates and supporting multifarious plant species, coupled with the islands' isolation, have facilitated the evolution of endemic species adapted to specific ecological conditions in the Bonin Islands. The role of adaptive radiation in driving the notable endemism rate of the Bonin Islands is exemplified by various genera, such as *Crepidiastrum* (Asteraceae) (Ito & Ono, 1990), *Symplocos* (Symplocaceae) (Soejima et al., 1994), and *Pittosporum* (Pittosporaceae) (Ito et al., 1997), which have experienced diversification yielding three to four species each.

In the Bonin Islands, the *Callicarpa* genus (Lamiaceae) is another example of adaptive radiation (Ono, 1991; Shimizu & Tabata, 1991). *Callicarpa* comprises approximately 140 perennial woody plants. This genus is found in temperate to tropical regions across East Asia, Southeast Asia, Australia, the Pacific Islands, and North and Central America. They are widely grown for their attractive appearance, particularly their bright, colorful berries, and are popularly known as “beautyberry.” In the Bonin Islands, *Callicarpa* includes three endemic species, *C. parvifolia*, *C. glabra*, and *C. subpubescens*. *Callicarpa parvifolia* and *C. glabra* are restricted to the Chichijima Islands, whereas *C. subpubescens* exhibits a wider distribution across the Mukojima and Hahajima Islands (Figure S1). Using microsatellite markers, Sugai et al. (2019) demonstrated genetic differentiation among the three *Callicarpa* species in the Chichijima Islands, as well as multiple genetic groups within *C. subpubescens* in the Mukojima and Hahajima Islands. Furthermore, Setsuko et al. (2024) identified four ecotypes in the Hahajima Islands, one derived from hybridization of two other ecotypes, with these ecotypes found to be locally adapted to specific soil moisture and light conditions. However, detailed phylogenetic relationships, as well as the diversification timing of species/ecotypes remains unclear. By examining differentiation patterns and divergence time, it may be possible to infer how plants in the oceanic islands diversified with geohistorical and/or environmental changes (Kadereit & Abbott, 2021).

In this study, we aimed to determine species and ecotype diversification patterns, estimate divergence times, and investigate cryptic species within the *Callicarpa* genus in the Bonin Islands. To achieve these objectives, we employed double‐digest restriction site‐associated DNA sequencing (ddRAD‐Seq), a genomic approach for detecting a large number of single nucleotide polymorphisms (SNPs) throughout the genome. Known for high resolution, ddRAD‐Seq is suitable for investigating intricate phylogenetic relationships within closely related taxa (Wagner et al., 2013). The results of this study will offer fundamental insights into the evolutionary history of *Callicarpa* in the Bonin Islands, contribute essential knowledge on factors influencing species diversification in island ecosystems, and enhance our understanding of speciation processes.

## 2 MATERIALS AND METHODS

### 2.1 Study species and sampling

*Callicarpa parvifolia* grows in sunny dry dwarf scrub on rocky ground in the Chichijima Islands (Toyoda, 2003), with a flowering peak in July (Table 1), whereas *C. glabra* grows in the understory of dry scrub in the Chichijima Islands (Toyoda, 2014) and has its flowering peak in August. These species are classified as “endangered” and “critically endangered” in the Red List of Threatened Species of Japan (Ministry of the Environment Government of Japan, 2020), respectively. In contrast, *C. subpubescens* is not listed as a threatened species and is widely distributed in the Bonin and Volcano Islands, situated approximately 150 km southwest of the Bonin Islands. *Callicarpa subpubescens* exhibits different ecotypes, each with distinct habitats and some with different flowering peaks. For example, the Chichijima Islands' ecotype (S) inhabits the forest edge of mesic forests, with peak flowering in June. The Hahajima Islands have four ecotypes: the glabrescent ecotype (SG), the tall ecotype (ST), the dwarf ecotype (SD), and the hybrid ecotype (SH; previously called the intermediate ecotype M; Setsuko et al., 2024). The ecotype SG inhabits the understory of mesic forests, with a flowering peak in July. The ecotype ST forms the canopy of tall mesic forests, with a flowering peak in October. The ecotype SD forms the canopy of dry scrub, with two flowering peaks in August and November. The ecotype SH forms the canopy of mesic scrub (cloud forests) or inhabits the forest edge of mesic forests, with a flowering peak in July (Setsuko et al., 2024; Sugai et al., 2019). The Mukojima Islands have two ecotypes (STm and Sm), which are genetically close to the ecotype ST in the Hahajima Islands and the ecotype S in the Chichijima Islands, respectively (Sugai et al., 2019; also refer to the Section 3). However, substantial forest areas of the Mukojima Islands have been lost due to feral goats (Shimizu, 2003), making it challenging to determine their original habitats. Flowering surveys on the Mukojima Islands were conducted only once in July 2010, as the islands are currently uninhabited, with no ocean liner services, and distant from inhabited Chichijima Island.

**TABLE 1:** Population characteristics of the three *Callicarpa* species in the Bonin Islands examined in this study.

Species	Island group	Island	Population Id^a^	Species/Ecotype^b^	No. individuals	Habitat	Tree size (average max. stem length, m)	Leaf hairs (ave. density of upper and lower surface/4 mm^2^)	Flowering peak
*C. parvifolia*	Chichijima	Anijima	Pa	P	7	Canopy of dry scrub	—	195.4, 558.0	—
		Chichijima	Pc	P	7		0.3	57.8, 556.9	Jul.^c^
*C. glabra*	Chichijima	Anijima	Ga	G	7	Understory of dry scrub	—	1.1, 2.8	—
		Chichijima	Gc	G	7		1.4	3.4, 2.6	Aug.^c^
*C. subpubescens*	Mukojima	Mukojima	STm	STm	7	—	—	17.5, 37.9	—
		Mukojima	Sm	Sm	7	—	—	7.1, 14.2	—
	Chichijima	Anijima	Sa	S	5	Edge of mesic forests	—	4.4, 19.0	—
		Chichijima	Sc	S	7		5.2	0.7, 15.6	Jun.^c^
	Hahajima	Hahajima	SGh	SG	7	Understory of mesic forests	3	0.9, 0.1	Jul.^d^
		Imoutojima	SGi	SG	6		—	0.7, 7.7	—
		Hahajima	SDh	SD	7	Canopy of dry scrub	1.5	21.8, 64.0	Aug. & Nov.^d^
		Imoutojima	SDi	SD	6		—	19.2, 38.5	—
		Hahajima	STh	ST	7	Canopy of mesic forests	7.1	22.3, 91.1	Oct.^d^
		Hahajima	SHh	SH	7	Canopy of mesic scrub or edge of mesic forests	3.5	17.5, 37.9	Jul.^d^

^a^ First letter is the initial letter of the species name, second letter is the ecotype abbreviation of the Hahajima and Mukojima Islands for *C. subpubescens*, and last lowercase letter is the initial letter of the island's name.

^b^ P: *C. parvifolia*; G: *C. glabra*; S: ecotype of *C. subpubescens* in the Chichijima Islands; SG: glabrescent ecotype of *C. subpubescens* in the Hahajima Islands; ST: tall ecotype of *C. subpubescens* in the Hahajima Islands; SD: dwarf ecotype of *C. subpubescens* in the Hahajima Islands; SH: hybrid ecotype of *C. subpubescens* in the Hahajima Islands; STm: ecotype of *C. subpubescens* similar to ecotype ST in the Mukojima Islands; Sm: *C. subpubescens* similar to ecotype S in the Mukojima Islands.

^c^ Sugai et al. (2019).

^d^ Setsuko et al. (2024).

To cover species and ecotypes of each island in the Bonin Islands, leaf samples were collected from 94 individuals across 14 populations for DNA extraction (Table 1, Figure S1). These samples included two populations (ecotypes STm and Sm) in the Mukojima Islands, six populations from the Chichijima Islands, representing three species and ecotypes (P, G, S) from two islands, Anijima and Chichijima Islands. Additionally, six populations from the Hahajima comprised two ecotypes (SG and SD) from the two islands, Hahajima and Imoutojima Islands, and ecotypes ST and SH from Hahajima Island. In the Hahajima Islands, leaf samples from Hahajima and Imoutojima Islands have been collected across the entire islands in our previous study (Setsuko et al., 2024). However, for the measurement of leaf morphology described below and to obtain high‐quality DNA, new samples were collected from the same populations. As outgroups, one individual each of *Callicarpa japonica* and *Callicarpa mollis*, both of which grow in Kyoto Prefecture, mainland Japan, was also collected (Figure S1). Silica gel was used to immediately dry leaf samples used for DNA extraction.

Leaves were also sampled for phenotypic measurements from the same individuals used for DNA extraction. As leaf morphology varies within individuals depending on sunlight exposure, leaves were collected from the sunlit upper canopy. However, owing to time constraints, sunlit leaves could not be collected from the SGi population.

### 2.2 Reference genome development

To obtain the reference genome of *C. subpubescens*, the individual with the highest homozygosity (based on previous studies using SSR markers, Setsuko et al., 2024) was selected from 51 individuals cultivated in a greenhouse at the Forestry and Forest Products Research Institute. DNA was extracted from fresh leaves using the Genomic‐tip (Qiagen, Germany). Library construction, using the SMRTbell Template Prep Kit (PacBio, USA), was performed according to the manufacturer's instructions. The DNA library was further fractionated using BluePippin (Sage Science, USA) to eliminate fragments <15 kb in size and sequenced using four single‐molecule real‐time cells on the Sequel system (PacBio, USA). DNA extraction, library preparation, and sequencing were conducted by the Kazusa DNA Research Institute (Chiba, Japan).

De novo genome assembly for *C. subpubescens* involved preprocessing to split chimera sequences using yacrd (Marijon et al., 2020). The assembly, conducted using wtdbg2 v. 2.5 (Ruan & Li, 2020), resulted in a genome size of approximately 450 Mb (Masuda et al., unpublished). The original dataset was approximately 81 × the size of the *C. subpubescens* genome. The dataset comprised a total of 482,624,924 bases and 6011 reads, with read lengths of 1129–5,172,107 bp (mean: 80,290 bp). The N50 sequence length was 623,636 bp. The quality of the assembly was assessed using the web tool gVolante (Nishimura et al., 2017). Using BUSCO (Simão et al., 2015) implemented in gVolante, approximately 86.1% of the complete core plant genes (1440 in total) were detected in the assembly.

### 2.3 ddRAD genotyping and SNP filtering

DNA was extracted using a modified CTAB method (Milligan, 1992). The DNA samples were quantified using a Qubit 2.0 Fluorometer (Invitrogen, MA, USA) and adjusted to 12.6 ng/μL through dilution with TE buffer. Sequencing libraries were prepared following a modified version of Peterson's protocol for ddRAD‐seq (Peterson et al., 2012). For detailed library preparation methods, refer to Appendix S1. The libraries were sequenced using a HiSeq2000 platform (Illumina, CA, USA) with 51‐bp single‐end reads at BGI Japan (Kobe, Japan).

To ensure appropriate data resolution and accuracy for each specific analysis, three datasets were created: denovo, referenced, and demography datasets. SNPs were detected using dDocent (Puritz et al., 2014) and Stacks version 2.60 (Catchen et al., 2011, 2013). The detection conditions and number of SNPs used in each analysis are summarized in Table S1. In all data sets, we excluded five individuals with low individual‐level genotyping rates from SNP detection. In the referenced and denovo datasets, SNPs were detected using dDocent, following its tutorial. When creating the denovo dataset, the reference genome of *C. subpubescens* was not used as a reference sequence and the two outgroup individuals were not included. This dataset is optimal for detecting population genetic structure without reference bias. In contrast, when creating the referenced dataset, the reference genome and two outgroup individuals were used. This dataset provides more accurate SNP calling for phylogenetic analysis. Total raw SNPs generated via dDocent were filtered using vcftools −0.1.14 to meet the conditions outlined in Table S1.

For the demography dataset, SNPs were re‐extracted from the .bam files created for the referenced dataset using dDocent. First, gstacks from Stacks was used to generate catalogs of variable sites (Rochette et al., 2019). Subsequently, populations from Stacks were employed to extract SNPs with the following options: ‐r 0.8 ‐p X ‐‐min‐mac 1 ‐‐max‐obs‐het 0.5 ‐‐vcf (where X represents the number of species/ecotypes in each dataset). The pairwise two‐dimensional minor allele site frequency spectrum (2D‐mSFS) was calculated from the .vcf file using the R script 2D‐msfs‐R (https://github.com/garageit46/2D‐msfs‐R). Missing data were addressed through bootstrapping within the same ecotype. This dataset includes non‐variable sites and low‐frequency SNPs, making it suitable for inferring evolutionary processes and population history.

### 2.4 Population genetic structure analysis

For individual‐based genetic structure analysis, the denovo dataset and ADMIXTURE program (Alexander et al., 2009) were used, with *K* values 1–15, as well as 30 iterations per *K* value, employed for analysis. The results were visualized using CLUMPAK (Kopelman et al., 2015), and the *K* value with the lowest cross‐validation (CV) error was considered the optimal *K*.

Neighbor‐net network analysis on the referenced dataset was conducted using SplitsTree4 (Huson & Bryant, 2006). Two analyses were performed: one including the ecotype SH suggested to originate from hybridization, and one excluding SH.

To reveal phylogenetic relationships of the *Callicarpa* genus in the Bonin Islands, the two individuals with the highest number of SNPs from each genetic group, as suggested by neighbor‐net network analysis, were selected. A maximum likelihood phylogenetic tree was constructed using RAxML‐NG v. 0.9.0 (Kozlov et al., 2019). We used the GTGTR4 + G + ASC_LEWIS model (GTR + Gamma model with an option to correct ascertainment bias), with 100 bootstraps. In RAxML‐NG analysis, phylogenetic trees were constructed using SNP datasets filtered at three different genotyping rates across all individuals: 30%, 50%, and 80%, to check if the topologies of the phylogenetic trees do not vary with different genotyping rates (Table S1).

### 2.5 Population demography inference

To identify divergence patterns among species/ecotypes of the *Callicarpa* genus in the Bonin Islands and their timing, a coalescent‐based maximum likelihood method was employed, estimating parameters of the population demographic model with fastsimcoal2 version 2705 (Excoffier et al., 2021). Ideally, we would analyze all recognized species/ecotypes at once to elucidate divergence patterns, but the complexity caused computational issues. Therefore, we divided the species/ecotypes based on our specific objectives. To elucidate divergence patterns, we excluded hybrid‐derived SH and the migratory ecotypes Sm and STm (see Section 3) focusing the analysis on six species/ecotypes. To estimate migration timing to the Mukojima Islands, we analyzed five ecotypes, including the two Mukojima ecotypes (Sm and STm), their presumed origins from Chichijima (S) and Hahajima (ST), and ecotype SG, which resembles Sm in appearance (see Section 3). Divergence models were sequentially applied for three to six ecotypes in the Chichijima and Hahajima Islands (Figure S2a–e) and from three to five ecotypes in the Chichijima, Hahajima, and Mukojima Islands (Figure S2f,g). Comparative divergence patterns for a model involving more than four‐ecotype divergences were designed based on the results from lower ecotype divergence modeling, admixture, phylogenetic tree, and neighbor‐net network analyses. Migration between species/ecotypes was only considered within the same island groups, as each island group was isolated by the sea, even during the Last Glacial Maximum (Setsuko et al., 2017). Only recent migration was assumed based on preliminary analyses; although ancient migration was also considered, such models exhibited much lower log‐likelihood values (data not shown). Details of each model are presented in Figures S3–S9.

The likelihood of each model was maximized from 50 random starting values, 40 expectation‐conditional‐maximization (ECM) optimization cycles, and 100,000 coalescent simulations. The mutation rate was set to 1.74 × 10^−8^, estimated in a woody species, *Populus tremula* (Gossmann et al., 2012). We considered the model with the lowest Akaike's information criterion value as the best model. The goodness of fitness of the best model was checked by visually comparing observed and simulated 2D‐mSFSs. The confidence interval of the best model was calculated via parametric bootstrapping. We simulated the model using fastsimcal2 with maximum likelihood estimate parameter values 100 times and obtained its 2D‐mSFSs. Using the simulated 2D‐mSFSs as input data, parameters of the best model were recalculated with the observed parameter values as a stating value, 15 ECM cycles, and 100,000 coalescent simulations. Finally, the 95% confidence interval (CI) was calculated from the obtained parameter values. Considering the ecological traits of our study species, 5 years per generation were used to convert an event time from generations ago to years ago, supported by cultivation experiments showing that ecotypes SD and SG flower within 1–2 years of sowing (Setsuko S., personal observation).

### 2.6 Leaf morphology

**FIGURE 1:**
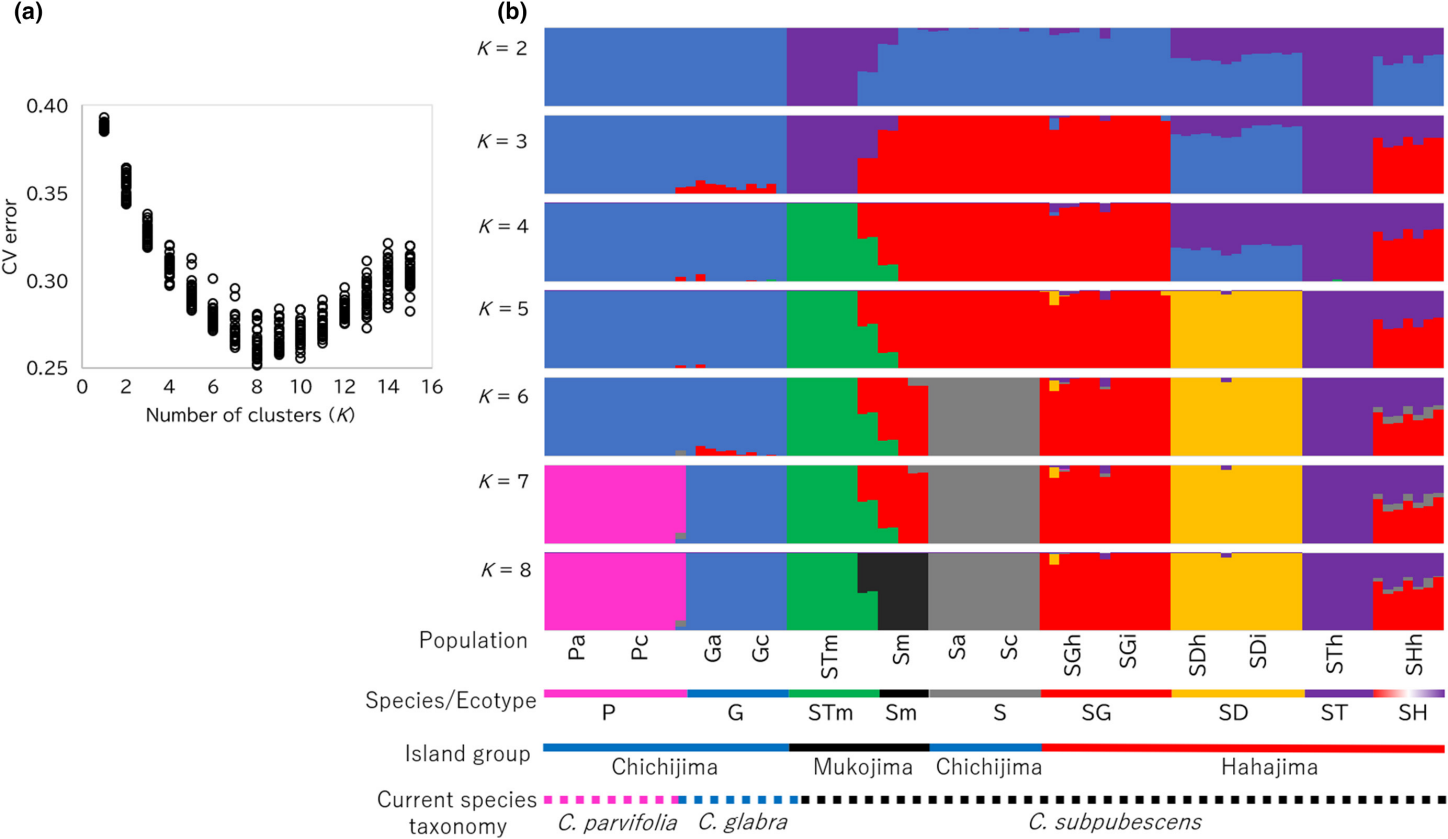
Results of ADMIXTURE analysis performed on 89 individuals from 14 populations of the Bonin Islands using denovo dataset. The cross‐validation (CV) error values for each run (a) and bar plots depicting the genetic admixture proportions for *K* = 2–8 (b). Vertical columns represent individuals; heights of bar plots are proportional to the posterior means of the estimated admixture proportions.

**FIGURE 2:**
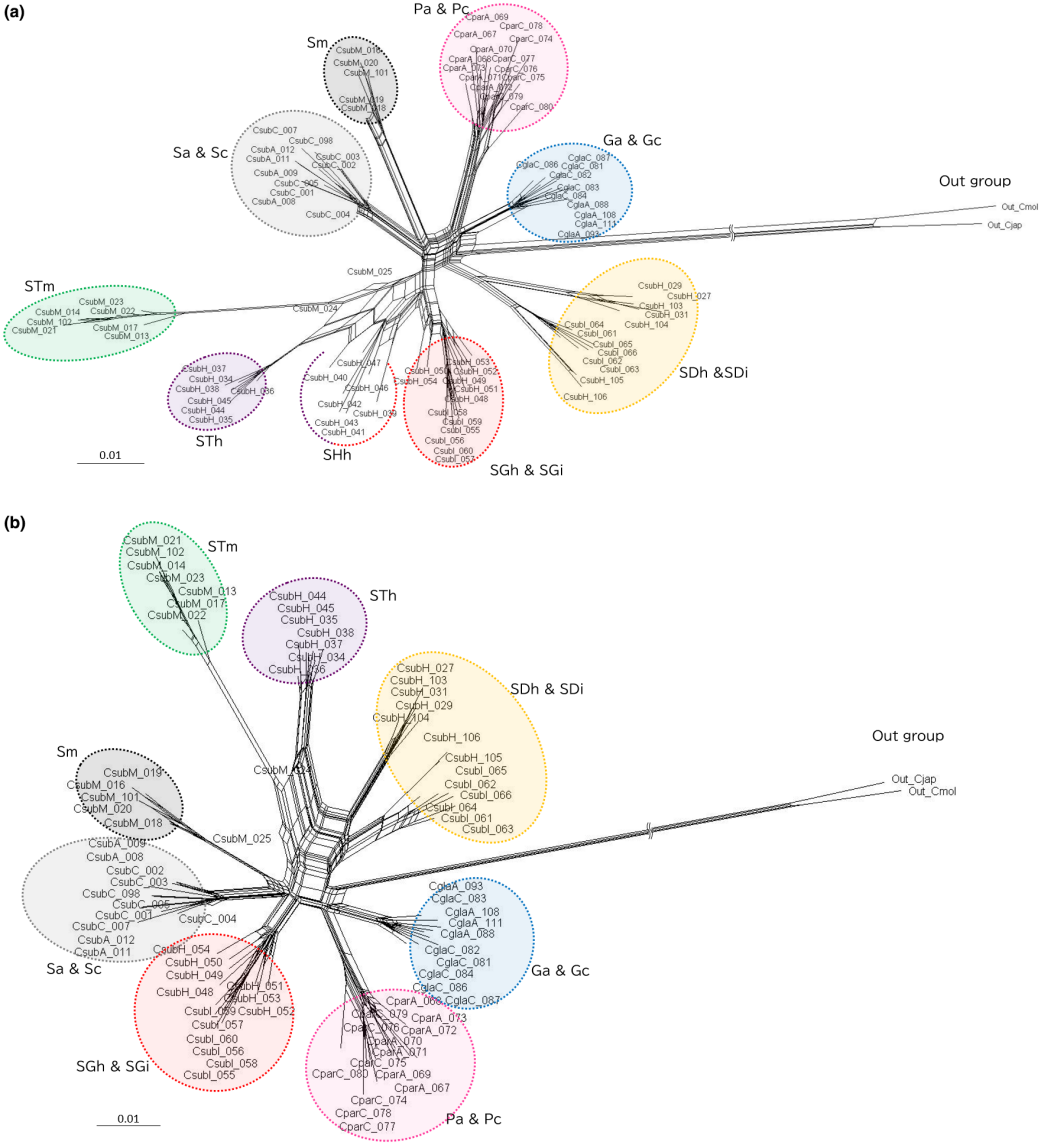
Neighbor‐net network tree reconstructed using 89 individuals from 14 populations (a) and 82 individuals from 13 populations (excluding ecotype SH, b) of the Bonin Islands and two outgroup individuals using the referenced dataset. Phylogenetic clade colors correspond to those used in Figures 1 and 3.

All individuals from the Bonin Islands were subjected to leaf phenotypic analysis, except population SGi, which lacked collected sunlit leaves. For each individual, one to five leaves were measured for the same 11 traits outlined by Setsuko et al. (2024): total length, blade length, blade width, hair density on the upper and lower surfaces of the leaf (i.e., number of hairs per 4 mm^2^), number of serrations per 30 mm, thickness of the leaf blade, leaf area, leaf mass per area, ratio of blade length to total leaf length, and ratio of leaf blade width to length. Principal component analysis (PCA) was performed on the measured traits. The two individuals from the Mukojima Islands that exhibited an admixed pattern were excluded from PCA (Figures 1 and 2).

## 3 RESULTS

### 3.1 Phylogenetic relationships

In ADMIXTURE analysis, the minimum CV error occurred at *K* = 8 (Figure 1a), revealing that the 14 populations in the Bonin Islands can be divided into eight genetic clusters: species P, species G, and ecotype S in the Chichijima Islands; ecotypes ST, SG, and SD in the Hahajima Islands; and ecotypes STm and Sm in the Mukojima Islands (Figure 1b). Ecotype SH on Hahajima Island exhibited a mixture of ecotypes ST and SG on these islands. Populations of the same species and ecotype from different islands within the same island groups shared the same genetic cluster (e.g., Pa & Pc, Ga & Gc, Sa & Sc, SGh & SGi, and SDh & SDi), whereas populations of the same ecotype from different island groups exhibited different genetic clusters (e.g., STh & STm, and Sa, Sc & Sm). When increasing the number of *K* from 2 to 8, the clusters that emerge were as follows: at *K* = 2, ecotypes ST and STm (purple) separated from other species and ecotypes (blue), while ecotypes SH and SD showed a mixture of purple and blue; at *K* = 3, ecotypes S, SG, and Sm (red) diverged from the blue cluster; at *K* = 4, ecotype STm (green) diverged from the purple cluster; at *K* = 5, ecotype SD (yellow) diverged from the mixture of purple and blue clusters; at *K* = 6, ecotype S (gray) diverged from the red cluster; at *K* = 7, species P (pink) diverged from blue cluster; finally, at *K* = 8, ecotype Sm (black) splits from the red cluster.

Neighbor‐net network analysis using SplitsTree revealed that the outgroups were positioned at the tips of exceptionally long branches (Figure S10). Fourteen populations in the Bonin Islands clustered into eight genetic groups, which exhibited star‐shaped patterns (Figure 2). Ecotype SH was scattered between ecotypes SG and ST, forming a reticulate structure (Figure 2a). Conversely, in the network diagram without ecotype SH, ecotype SG was located between ecotype S and species P, rather than being close to ecotype ST, suggesting that ecotypes SG and ST were not genetically similar (Figure 2b). Akin to the results from ADMIXTURE analysis, populations of the same ecotype from different islands within the same island groups shared the same genetic groups, whereas populations of the same ecotype from different island groups exhibited different genetic groups. The ADMIXTURE analysis and neighbor‐net network analysis suggest the presence of genetic groups derived from hybrids (ecotype SH) between ecotypes SG and ST. When discussing the evolutionary process within *Callicarpa* in the Bonin Islands, these hybrid groups should be excluded from phylogenetic analysis.

**FIGURE 3:**
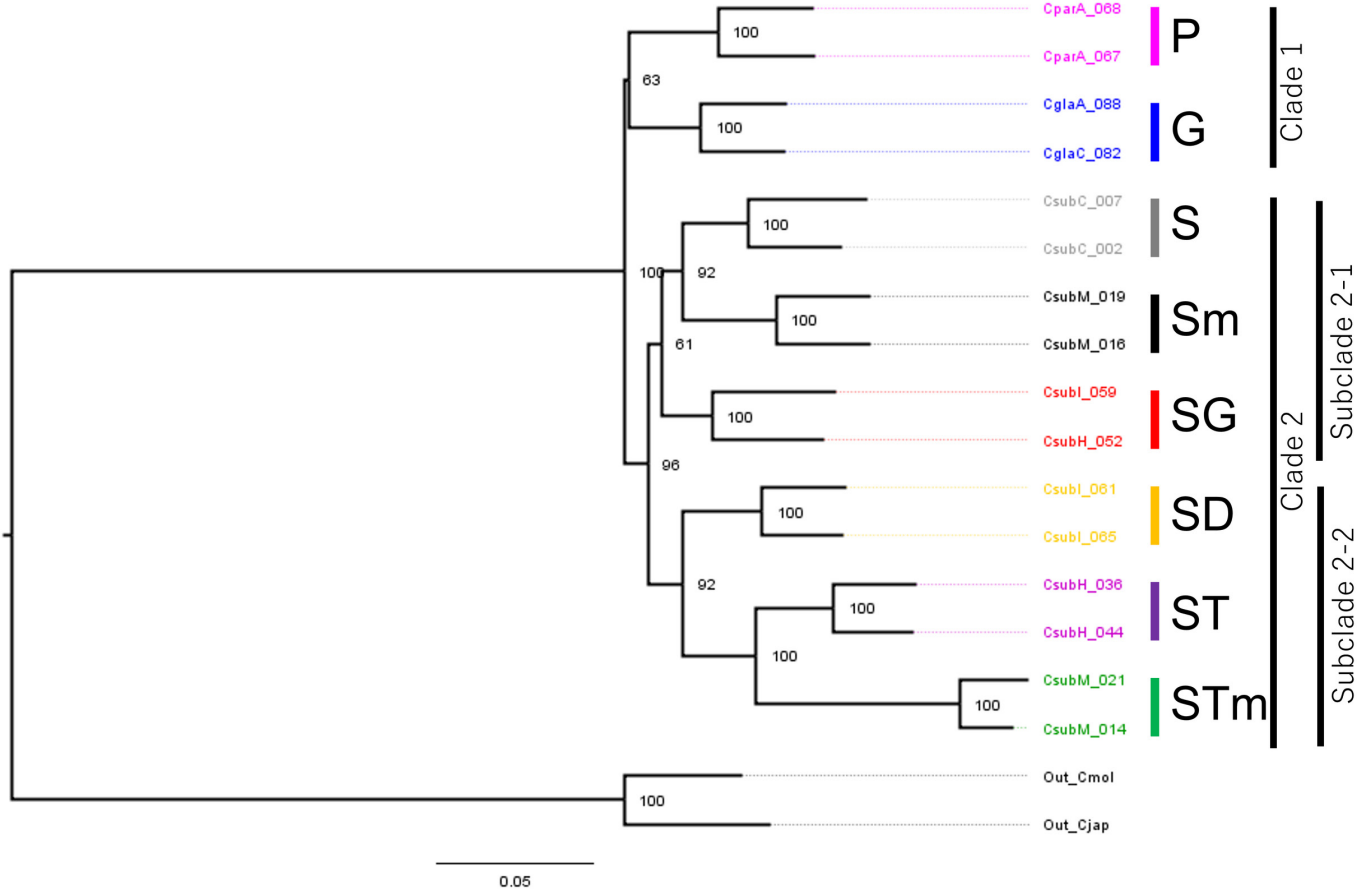
The phylogenetic tree inferred from RAxML‐NG utilizes SNPs with a genotyping rate of 80% from a referenced dataset comprising a total of 16 individuals (excluding ecotype SH) from eight genetic groups in the Bonin Islands, and two individuals of the outgroup (Out_Cmol, Out_Cjap). Phylogenetic clade colors correspond to those used in Figures 1 and 2.

The phylogenetic trees generated by RAxML‐NG without ecotype SH showed consistent tree topologies across datasets with different genotyping rates (Figure S11). However, some branches had low support values, with bootstrap probabilities below 70% (e.g., the branches between clades P & G, and between the clade consisting of S & Sm and the clade SG). In the phylogenetic trees, eight species/ecotypes in the Bonin Islands were divided into clade 1 (species P and G) and clade 2 (ecotypes of *C. subpubescens*) (Figure 3). Clade 1 was further divided into two subclades, with species P and G, as monophyletic clades. Clade 2 was divided into subclade 2–1 (ecotypes S, Sm, and SG) and subclade 2–2 (ecotypes SD, ST, and STm).

### 3.2 Inference of population demography

**FIGURE 4:**
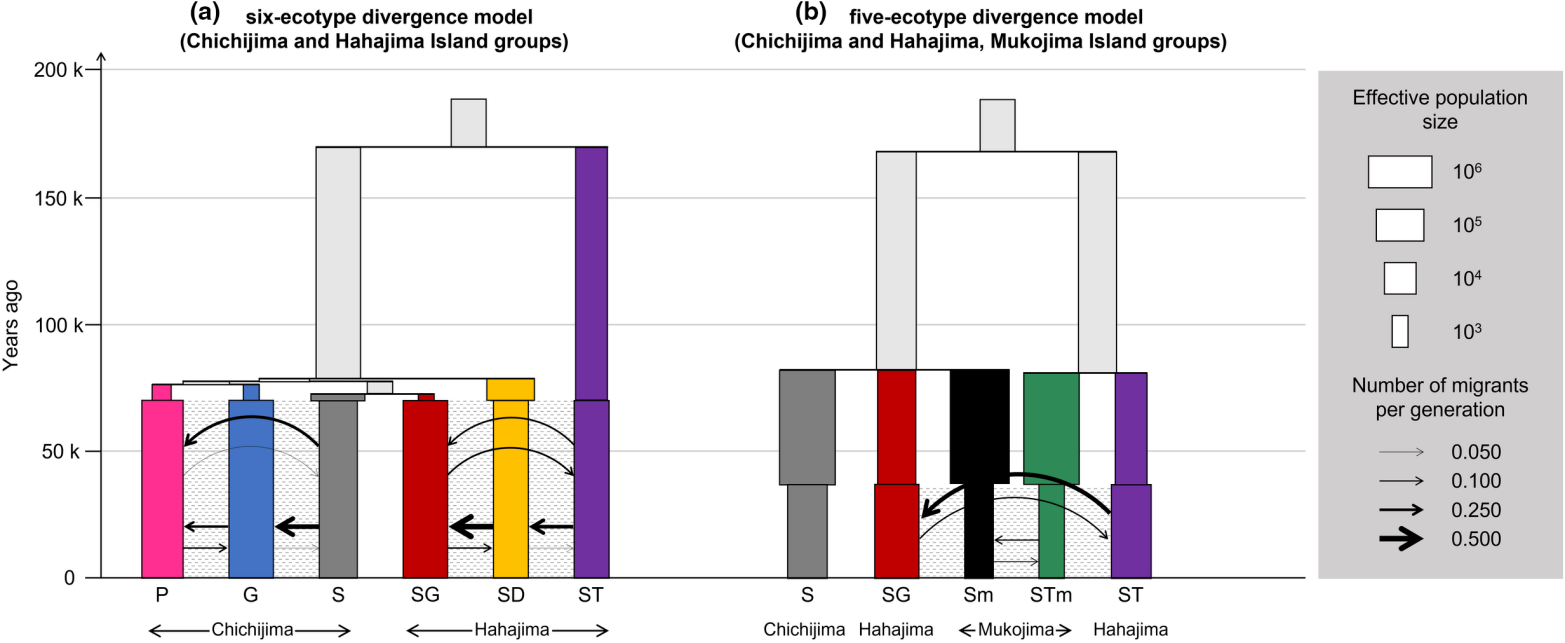
Best six‐species/ecotype divergence model (a), and best five‐ecotype divergence model (b). These correspond to models e1 and g2 in Figure S2, respectively. Period shown in dashed lines assumes migration between ecotypes. Direction of migration shows the movements of individuals (i.e., forward‐in‐time). Ecotype colors correspond to those used in Figures 1, 2, 3.

Regarding divergence patterns among the six species/ecotypes (species P, species G, and ecotype S in the Chichijima Islands; and ecotypes ST, SG, and SD in the Hahajima Islands), at step (a), among the three‐species divergence models in the Chichijima Islands, model a2 was the best model (Figure S2a, Table S2). At step (b), among the four‐species/ecotype divergence models in the Chichijima and Hahajima Islands, model b1 was the best model (Figure S2b, Table S3). Similarly, at steps (c) and (d), among the three‐ and four‐species/ecotype divergence models in the Hahajima Islands and in the Chichijima and Hahajima Islands, models c2 and d2 were the best models (Figure S2c,d, Tables S4 and S5). Among six‐species/ecotype divergence models in the Chichijima and Hahajima Islands, built considering steps (a)–(d), model e1 was the best model (Figure S2e, Table S6). The model e5, showing the same branching pattern as the RAxML‐NG phylogenetic tree without ecotype SH (Figure 3), had a considerably higher AIC compared to other models, indicating it is less suitable. Therefore, we decided to adopt model e1 for the divergence patterns among the six species/ecotypes in the Chichijima and Hahajima Islands. In model e1, most species/ecotypes, except for ecotypes S and SD, exhibited significant recent population expansion at 70.2 kya (95% CI could not be estimated; refer to Table S7), whereas S and SD did not show significant population size change (Figure 4a, Tables S7 and S8). Divergence times *T*_2_–*T*_5_ showed a narrow range of 72.9–76.9 kya, implying that most species/ecotypes underwent simultaneous divergence. However, the divergence time between ecotype ST and the other ecotypes was 170.7 (95% CI: 162.2–183.4) kya, indicating a much earlier divergence for ecotype ST.

Regarding the divergence patterns of the five ecotypes in the Chichijima, Hahajima, and Mukojima Islands, at step (f), among the three‐ecotype divergence models in these islands, model f1 was the best model (Figure S2f, Table S9). At step (g), among the five‐ecotype divergence models in the Chichijima, Hahajima, and Mukojima Islands, model g2 was the best model (Figure S2g, Table S10). Therefore, we decided to adopt model g2 for the divergence patterns among the five ecotypes in the Chichijima, Hahajima, and Mukojima Islands. In this model, ecotype S and the two ecotypes in Mukojima Islands (Sm and STm) showed significant recent population reduction at 37.1 (95% CI: 33.9–40.5) kya, whereas the two ecotypes in the Hahajima Islands (SG and ST) exhibited significant recent population expansion (Figure 4b, Tables S8 and S11). Divergence times *T*_2_ and *T*_3_ were 81.4 (72.7–86.4) and 82.3 (75.0–88.2) kya, respectively, and were very close, suggesting recent and possibly synchronized divergence events in the Mukojima Islands. However, the divergence time between the two ancestral lineages was 168.8 (95% CI: 160.6–179.1) kya.

Migrant numbers per generation estimated in the best models e1 and g2 were 0.020–0.511 and all significantly lower than 1.0 (Tables S7 and S11). These two best models, e1 and g2, indicated that, although the timings of recent population size change and the onset of migration differed between models, the timings of divergence between ecotypes were very similar (approximately 80 and 170 kya; Figure 4, Tables S7 and S11), that is, most ecotypes underwent recent divergence (approximately 73–77 kya), whereas ST experienced ancient divergence (around 170 kya).

### 3.3 Leaf morphology

**FIGURE 5:**
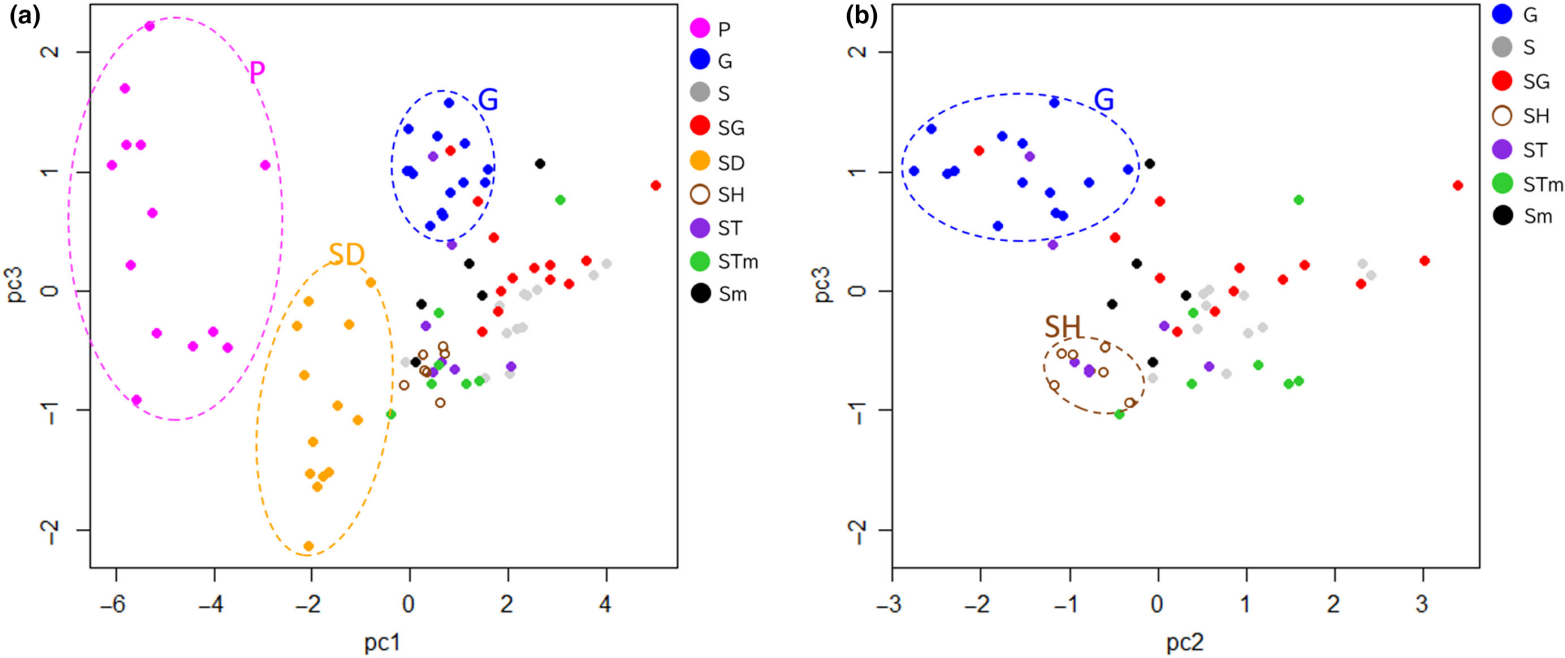
Distributions of the first and third principal components (pc1 and pc3) for 11 leaf morphological traits of nine species/ecotypes (a), and those of the second and third principal components (pc2 and pc3) for ecotypes of 11 leaf morphological traits of seven species/ecotypes (ecotypes P and SD are masked) (b).

In PCA of leaf morphologies, species P, species G, and ecotype SD exhibited distinct distributions (Figure 5). Most *C. subpubescens* ecotypes did not show independent distributions, whereas ecotype SH showed a relatively narrow distribution but was included in the ecotype ST distribution. The leaves of species P were characterized as small, round, remarkably hairy, and thick (Figure S12). Ecotype SD also showed small, round leaves, similar to those of species P, but with a significantly lower hair mass and smaller leaf thickness. Species G possessed small, elongated, and moderately thick leaves lacking hair. Ecotype S is identical to the registered type specimen of *C. subpubescens* (specimen no. K000674714), exhibiting fine, soft hairs on its leaves, whereas ecotype SG has almost no hairs on its leaves. However, the number of hairs did not differ compared with ecotype SG (Figure S12), and the plot distributions of ecotypes S and SG overlapped in PCA (Figure 5). Ecotypes SH and ST possessed similar leaves, with ecotype SH occupying part of the broader distribution of ecotype ST in the PCA plot. However, the plot distributions of ecotypes SH and SG in PCA did not overlap.

## 4 DISCUSSION

### 4.1 Phylogenetic relationships and divergence patterns

In the three *Callicarpa* species in the Bonin Islands, our results revealed that the divergence of species and ecotypes occurred relatively recently, around 70–170 thousand years ago, almost simultaneously. Oceanic island biotas often experience frequent ecological speciation and adaptive radiation. Our study, which demonstrates simultaneous species and ecotype differentiation in *Callicarpa* using a large number of genome‐wide SNPs, is considered important and conclusive evidence of adaptive radiation in the Bonin Islands.

On the other hand, contradictions were observed among the analyses conducted in this study. For example, the neighbor‐net tree and phylogenetic tree show that species P and G are closest to the outgroup and are more ancestral. In contrast, ADMIXTURE and demographic analyses using fastsimcoal2 suggest that ecotype ST is closer to the ancestral species, leading to an apparent conflict. Masuda et al. (submitted) investigated the phylogenetic relationships among *Callicarpa* species in East Asia and three *Callicarpa* species in the Bonin Islands using different datasets, including whole chloroplast genomes, 86 single‐copy genes, and over 10,000 nuclear genes (whole nuclear genome). The divergence patterns of the three *Callicarpa* species in the Bonin Islands varied among datasets, and a consistent trend was not observed. The lack of consistent results in the divergence patterns of this genus in the Bonin Islands across analyses and datasets is not due to inappropriate analysis methods but might be attributed to insufficient accumulation of mutations for species and ecotypes and/or incomplete lineage sorting among them due to rapid radiation (Pollard et al., 2006).

It cannot be definitively stated that the ecotype ST is the most ancient lineage, as this is not supported by the neighbor‐net tree and phylogenetic trees. However, it is likely that other species and ecotypes underwent rapid diversification within a short period, as indicated by demographic analysis and the star‐shape of neighbor‐net tree (Suh et al., 2015). The simultaneously diverged species and ecotypes include species P and ecotype SD which adapted to dry environments, and species G and ecotype SG which adapted to understory forests. This result suggests that new environments, such as dry areas and lower height forests, emerged in the islands, prompting rapid adaptive radiation. The demography model suggests that simultaneous divergence occurred 73–77 kya. Given that 73–77 kya, corresponds to the time when Marine Isotope Stage (MIS) 5 (interglacial period) changed to MIS 4 (glacial period) (Martinson et al., 1987), estimated to have been a period of rapid cooling, cold weather may have triggered the simultaneous divergence of this taxon. Kadereit and Abbott (2021) reviewed studies examining divergence times from phylogenetic trees from all continents and major climatic zones, finding that many plant speciation events occurred in the Quaternary and suggesting that climate change during this period was the cause.

Our study suggests species and ecotypes adapted to a dry environment (species P and ecotype SD) and forest understory and forest edge environments (species G and ecotypes S and SG) diverged at the same time from ecotype ST that constitutes the canopy of tall mesic forests. Although speciation timing may lack precision due to methodological challenges regarding glacial or interglacial periods, it is evident that simultaneous diversification occurred. Considering the characteristics of the divergent species/ecotypes, the timing of speciation likely aligns with the onset of aridification on the islands. Species/ecotypes adapted to the forest understory and forest edge environment may seem unrelated to aridification. However, species G inhabits dry scrub (Toyoda, 2014), ecotype S inhabits mesic forest edges, and ecotype SG does not flower in the darkest tall forests (Setsuko S., personal observation). Therefore, they are all considered maladapted to taller forests where ecotype ST grows. This suggests that the environment has changed from tall forests to shorter forests with more forest edges (Olson et al., 2018), potentially caused by the aridification of the islands.

Examples of organisms rapidly altering their phenotypes upon aridification have been reported in animals, such as Darwin's finches (Grant & Grant, 2006), and in plants, such as *Mimulus* and *Brassica* (Dickman et al., 2019; Johnson et al., 2022). Selection has also been observed on the *HMGA2* gene, causing beak size variation during drought in Darwin's finches (Lamichhaney et al., 2016), and multiple genes associated with drought response traits evolving during drought in *Brassica* (Franks et al., 2016; Johnson et al., 2023). In the *Callicarpa* genus in the Bonin Islands, rapid adaptation to aridification may have led to speciation. Therefore, future research will involve identifying genes associated with drought adaptation.

Surprisingly, we revealed that the differentiation timing of the five species/ecotypes, occurring 73–77 kya, and the migration of ecotypes Sm and STm in the Mukojima Islands from ecotypes S in the Chichijima Islands and ST in the Hahajima Islands, respectively, took place during approximately the same period, around 81–82 kya (Figure 4, Tables S7 and S11). Aridification usually hinders the successful fruit reproduction of previously abundant plants (Abobatta, 2021), leading to food shortages across the entire island; therefore, it is plausible that avian seed dispersers may have moved to new islands in search of food (Boyle & Conway, 2007).

Population size changes, exhibiting large increases of more than two orders, were observed for species/ecotypes P, G, and SG in model e1 (Figure 4a, Tables S7 and S8). Dry scrub, the habitat of species P and G, may have increased due to aridification during the glacial period. Ecotype SG is suggested to have originated from ecotype S in the Chichijima Islands and migrated to the Hahajima Islands, based on the phylogenetic tree and demographic analysis. Ecotype SG currently inhabits the understory of mesic forests throughout the Hahajima Islands and has the largest population of any ecotype in the Hahajima Islands (Setsuko et al., 2024). Compared with the Chichijima Islands, the area of mesic forests is larger in the Hahajima Islands (Shimizu, 1999), and the substantial increase in population size of ecotype SG may be due to successful adaptation to the mesic forest environment through migration from the Chichijima Islands to the Hahajima Islands. However, large decreases of more than two orders were observed in ecotype Sm and STm in model g2 (Figure 4b, Tables S8 and S11). This may be due to the limited number of individuals that migrated from the original island populations to the Mukojima Islands (i.e., the founder effect).

### 4.2 Cryptic species

Phenotypic and genetic differences, as well as diversification patterns, were used to assess current species classifications. *Callicarpa parvifolia* and *C. glabra* not only differ in their habitats but also show distinct differences in leaf morphology (Table 1, Figure 5). Phylogenetically, these two species form a sister clade, each maintaining monophyly (Figure 3). Accordingly, the current taxonomy of the two species appears reasonable.

Ecotype SD forms a canopy in dry scrub, with leaves similar to *C. parvifolia* (Figure S12). However, it can be distinguished from *C. parvifolia* by significantly lower leaf hair density and thickness. Phylogenetically, ecotype SD and *C. parvifolia* were located on different clades (Figure 3). A parallel evolutionary process of adaptation to dry environments likely resulted in a morphology closely resembling that of *C. parvifolia*. Additionally, compared to ecotype S from the Chichijima Islands, which is identical to the registered type specimen of *C. subpubescens*, ecotype SD differs both in its leaf morphology and flowering phenology, with prolonged flowering from summer to winter (Table 1), warranting recognition as a new species.

Ecotype ST is grouped in the same subclade as ecotype SD in the phylogenetic tree (Figure 3), however, they can be distinguished based on leaf morphology (Figure 5). Among ecotype ST and other ecotypes (S, SG, and SH), they cannot be distinguished based solely on leaf morphology; however, ecotype ST can be distinguished from other ecotypes using flowering phenology differences (the flowering season of ecotype ST is autumn, whereas those of ecotypes S, SG, and SH are summer; Table 1). Therefore, we consider ecotype ST to be a cryptic species.

Both ecotype S in the Chichijima Islands and ecotype SG in the Hahajima Islands flower in summer (Table 1). Ecotype S and SG are grouped in the same subclade, and no obvious phenotypic differences were observed. Several examples exist in the Bonin Islands of the same species differentiated genetically due to gene flow restriction caused by the different island groups they inhabit (Setsuko et al., 2017, 2020, 2022; Sugai et al., 2013). Therefore, considering ecotype SG as homologous to ecotype S is reasonable.

Ecotype SH, thought to be derived from a hybrid of ecotypes ST and SG, is found only on Hahajima Island (Figures 1b and 2a). As the flowering season of ecotypes SG and SH is summer and that of ecotype ST is autumn (Table 1), ecotypes SH and ST are distinguished by their flowering periods, whereas ecotypes SH and SG are roughly distinguished by their leaf morphologies. In naturally distributed individuals, habitat information can aid taxonomic classification of ecotypes SG and SH, as ecotype SH inhabits high‐elevation cloud forests and forms the forest canopy layer, whereas ecotype SG inhabits the understory of mesic forests, except in the high‐elevation areas of the Hahajima Island (Setsuko et al., 2024). Based on these findings, we propose that *C. subpubescens* can be divided into three species in addition to one hybrid‐derived taxon, rather than one species.

Concerning ecotypes in the Mukojima Islands, the flowering of STm and Sm was investigated only once in July, with these ecotypes found to be in the early and late stages of flowering, respectively. July marked the beginning of flowering for ecotype ST in the Hahajima Islands, and the end of flowering for ecotype S in the Chichijima Islands (Table 1). Combining flowering information with the phylogenetic tree results from this study, Sm was considered the same ecotype as S, whereas STm was considered the same ecotype as ST. Determining whether ecotypes STm and Sm in the Mukojima Islands can be considered identical to ecotypes ST and S, respectively, necessitates a more thorough investigation into the flowering periods of ecotypes STm and Sm. Furthermore, although only a minimal number of hybrid individuals (CsubM_024 and Csub_025) were found in the Mukojima Islands (Figures 1b and 2), the extent to which they form hybrid zones, akin to ecotype SH on Hahajima Island (Setsuko et al., 2024), remains unclear. However, given the coexistence of lineages from different origins on the same island, there is a possibility that this codistribution contributes to hybrid speciation (Kagawa & Takimoto, 2018). Further research should include more comprehensive investigations into hybridization in the Mukojima Islands.

### 4.3 Long seed dispersal between the island groups

In phylogenetic tree analysis, ecotypes S in the Chichijima Islands, SG in the Hahajima Islands, and Sm in the Mukojima Islands together with subclade 1–2, whereas ecotypes ST in the Hahajima Islands and STm in the Mukojima Islands formed another subclade (Figure 3), indicating potential interisland group migration. The primary seed disperser of *Callicarpa* is the brown‐eared bulbul (*Hypsipetes amauroti*s). Furthermore, metabarcoding from the feces of the Japanese wood pigeon (*Columba janthina nitens*), endemic to the Bonin Islands, occasionally showed the presence of *Callicarpa* seeds (Ando et al., 2016). Although pigeons are not considered efficient seed dispersers due to seed crushing in their gizzards, seeds up to 3 mm in size remain intact in their feces (Shibazaki & Hoshi, 2006). Additionally, until 1920, the jungle crow (*Corvus macrorhynchos*) was present in the Bonin Islands (Higuchi, 1984). Estimated seed dispersal distances for these bird species, based on body size (Dunning Jr., 2007), and calculated using a phylogenetic generalized least squares model for seed retention time (Yoshikawa et al., 2019) and flight speed (Tennekes, 2009), are as follows: brown‐eared bulbul, 39.7 km; Japanese wood pigeon, 88.8 km; and jungle crow, 110 km. The current distances between the Mukojima and Chichijima, Chichijima and Hahajima, and Mukojima and Hahajima Islands are 32, 35, and 110 km, respectively. The presence of the same ecotypes in different island groups is likely a result of long‐distance seed dispersal by these birds. Particularly, ecotype ST, inhabiting the mesic forests of the Hahajima Islands, occurs in the Mukojima Islands but not in the Chichijima Islands, despite similar mesic forests in the latter. It is speculated that ecotype ST migrated between the Hahajima and Mukojima Islands through birds with high flight ability, bypassing the Chichijima Islands. However, due to genetic differentiation even among the same ecotypes in different island groups, the occurrence of long‐distance seed dispersal between islands is expected to be extremely rare.

## 5 CONCLUSION

Our study on the *Callicarpa* genus in the Bonin Islands revealed that the ancestral species of this genus, which arrived on small oceanic islands adapted to different environments simultaneously very recently over a very short period, demonstrating a typical case of adaptive radiation. The concurrent diversification of species/ecotypes adapted to different environments suggests a connection to island aridification. Shifts from tall forests to lower forests with increased forest edges and an increase in dry environments likely triggered rapid phenotypic and genetic changes for adaptation, as observed in other organisms. Future research will focus on identifying genes associated with drought adaptation. Cryptic species were found within *C. subpubescens*, with ecotypes SD and ST considered distinct species based on leaf morphology, flowering phenology, and phylogenetic patterns. Although genetically distinct, ecotypes S and SG show no significant phenotypic differences and can be treated as the same species. Ecotype SH, presumed to be a hybrid between ecotypes ST and SG and found only on Hahajima Island, exhibited different characteristics from its parent ecotypes. Long‐distance seed dispersal events likely contributed to the presence of the same ecotypes in different island groups. Certain birds, such as the brown‐eared bulbul, Japanese wood pigeon, and jungle crow, are potential seed dispersers and contribute to rare long‐distance seed dispersal.

## AUTHOR CONTRIBUTIONS

**Suzuki Setsuko:** Conceptualization (lead); data curation (lead); formal analysis (lead); funding acquisition (equal); investigation (equal); methodology (lead); project administration (lead); supervision (equal); visualization (lead); writing – original draft (lead); writing – review and editing (lead). **Satoshi Narita:** Conceptualization (equal); data curation (equal); formal analysis (equal); investigation (lead). **Ichiro Tamaki:** Data curation (supporting); formal analysis (supporting); methodology (equal); visualization (equal); writing – original draft (supporting). **Kyoko Sugai:** Conceptualization (supporting); investigation (supporting); validation (equal). **Atsushi J. Nagano:** Formal analysis (supporting); investigation (equal); methodology (equal); writing – original draft (supporting). **Tokuko Ujino‐Ihara:** Data curation (supporting). **Hidetoshi Kato:** Conceptualization (equal); funding acquisition (equal); investigation (equal); project administration (equal); supervision (equal). **Yuji Isagi:** Conceptualization (equal); funding acquisition (equal); project administration (equal); supervision (equal).

## FUNDING INFORMATION

This work was funded by Grants‐in‐Aid for Scientific Research from the Japanese Society for Promotion of Science (JP26290073, JP15K07203, JP21K05694, JP24K01801), the Environment Research and Technology Development Fund of the Environmental Restoration and Conservation Agency provided by Ministry of the Environment of Japan (JPMEERF20144002, JPMEERF20224M02).

## CONFLICT OF INTEREST STATEMENT

Authors declare no competing interests.

## Supporting information


Appendix S1.

## Data Availability

Genotype data have been deposited at FigShare: https://doi.org/10.5061/dryad.05qfttfc1. The PacBio Sequel raw reads are available at NCBI Sequence Reads Archive (DRA017215), and the reference genome sequence of *C. subpubescens* has been deposited at DDBJ/EMBL/GenBank under the accessions BTTA01000001–BTTA0106011.
