# Supplementary material for: Adaptive radiation of the Callicarpa genus in the Bonin Islands revealed through double‐digest restriction site‐associated DNA sequencing analysis
Source: Ecol Evol. 2024 Sep 13;14(9):e70216. doi: 10.1002/ece3.70216 (PMC11393766; doi:10.1002/ece3.70216)
Supplement: Supplementary file 1 — Appendix S1. [file ECE3-14-e70216-s001.docx]

**Appendix 1** Detailed methods of ddRAD library preparation.

Genomic DNA fragments were fragmented by treating 10 ng of the DNA with EcoRI and BglII enzymes. The digestion and subsequent ligation steps were carried out in a 10 μL reaction volume at 37 °C for 16 h. The reaction mixture included 20–40 ng of genomic DNA, 0.5 μL of each 10U/μL EcoRI and BglII enzymes (Takara, Kyoto, Japan), 1 μL of ×10 NEB buffer 2, 0.1 μL of ×100 BSA (New England Biolabs, Ipswich, USA), 0.4 μL of each 5 μM EcoRI and BglII adapters, 0.1 μL of 100 mM ATP, and 0.5 μL of T4 DNA ligase (Enzymatics, Beverly, USA). The ligated product was subsequently purified using AMPure XP beads (Beckman Coulter, Brea, USA). The purified DNA, now ligated with adapters, was then subjected to PCR amplification. The PCR reaction was carried out in a 10 μL reaction volume containing 2 μL of the adaptor-ligated DNA, 2 μL of 5 μM index primer with 6-mer variable sequences for sample identification, 1 μL of 10 μM TruSeq universal primer, and 5 μL of ×2 KAPA HiFi HotStart ReadyMix (KAPA Biosystems, Wilmington, USA). The PCR process involved initial denaturation at 94 °C for 4 m, followed by 20 cycles of 10 s at 98 °C, 15 s at 65 °C, and 15 s at 68 °C. The PCR products obtained from different samples were combined and then subjected to a further round of purification using AMPure XP beads. Subsequently, DNA fragments ranging in size from 350 to 400 bp were isolated from the purified DNA solution using electrophoresis on a 2.0% E-Gel SizeSelect gel (Life Technologies, Carlsbad, USA). The quantification of the DNA was performed using the QuantiFluor dsDNA System (Promega, Madison, USA), while the quality assessment was carried out using an Agilent 2100 Bioanalyzer (Agilent Technologies, Santa Clara, USA).


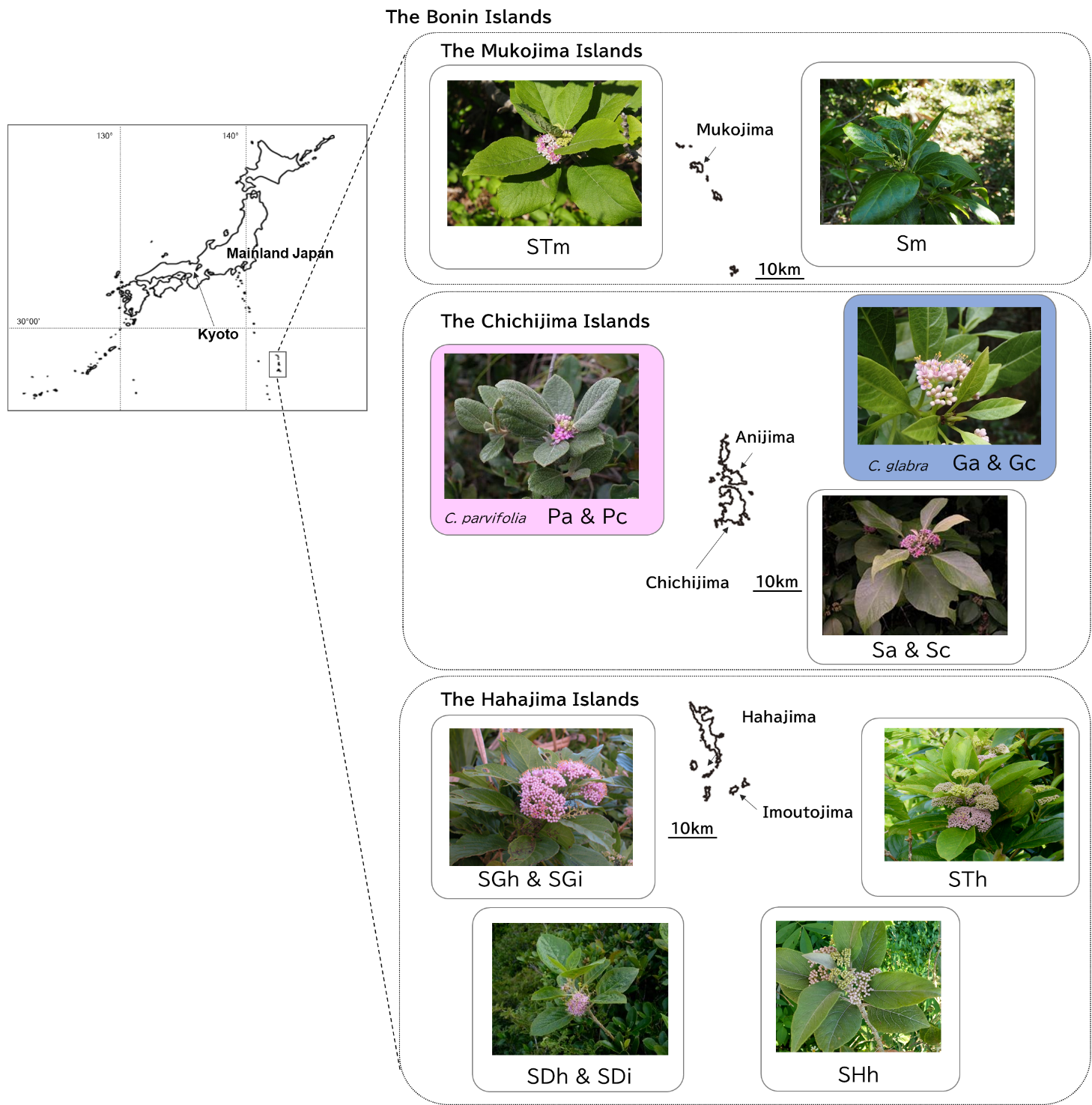


**Fig. S1** The locations of the Mukojima, Chichijima, and Hahajima Islands within the Bonin Islands and the names of the populations used in the analysis. Pink frame indicates *Callicarpa parvifolia*, blue frame indicates *C. glabra*, and white frames are all classified as *C. subpubescens* in current taxonomy. The letters in the frames indicate the population IDs in Table 1.

**
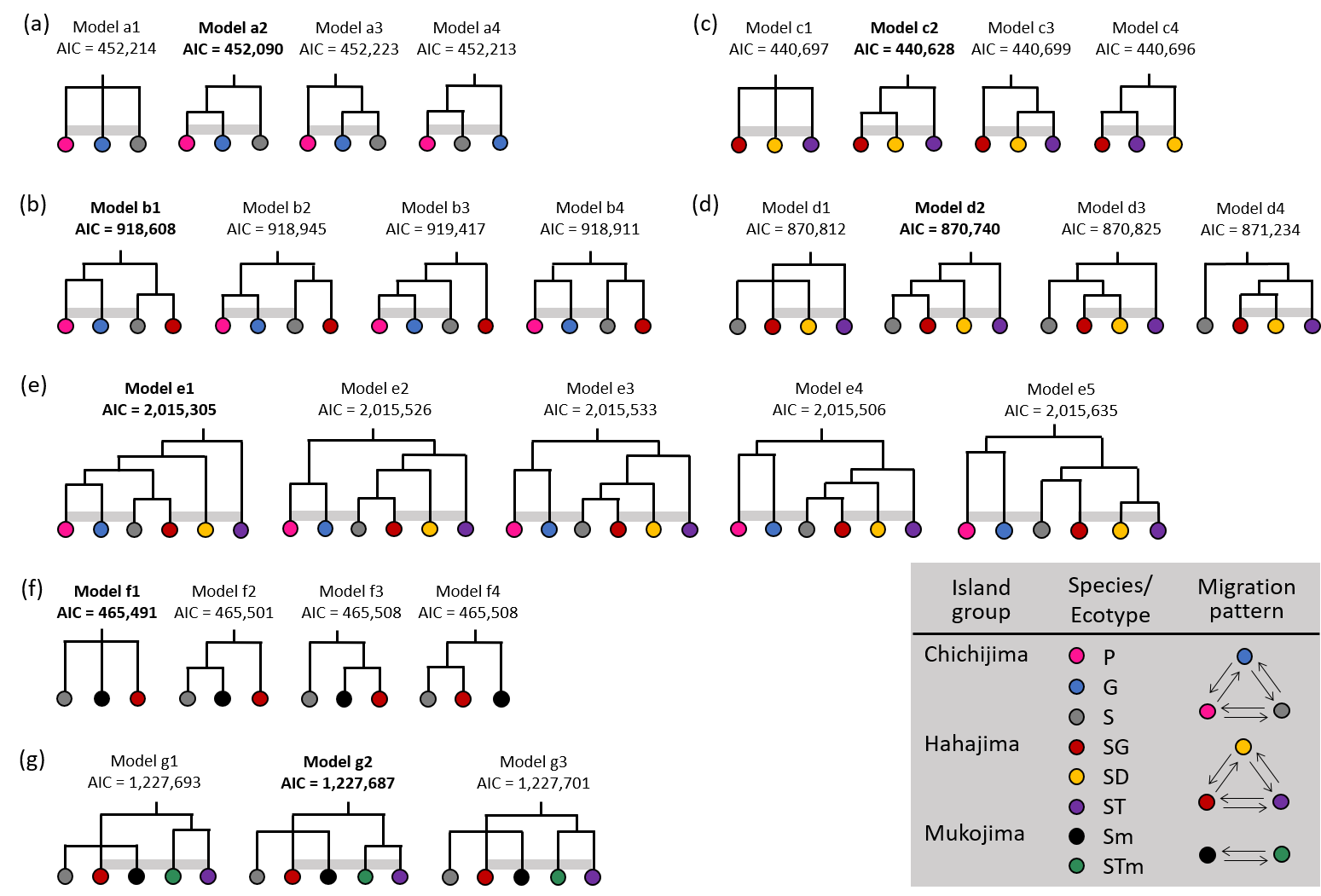
**

**Fig. S2** Compared population demographic models and their Akaike’s information criterion (AIC) values. (a) Three-species divergence models (three Chichijima species); (b) four- species/ecotype divergence models (three Chichijima and one Hahajima species/ecotypes); (c) three-ecotype divergence models (three Hahajima ecotypes); (d) four-ecotype divergence models (one Chichijima and three Hahajima ecotypes); (e) six- species/ecotype divergence models (three Chichijima and three Hahajima species/ecotypes); (f) three-ecotype divergence models (one Chichijima, one Hahajima and one Mukojima ecotypes); (g) five-ecotype divergence models (one Chichijima, two Hahajima and two Mukojima ecotypes). The gray-colored horizontal bars between branches assume migration between species/ecotypes. The best model is shown in bold.

**
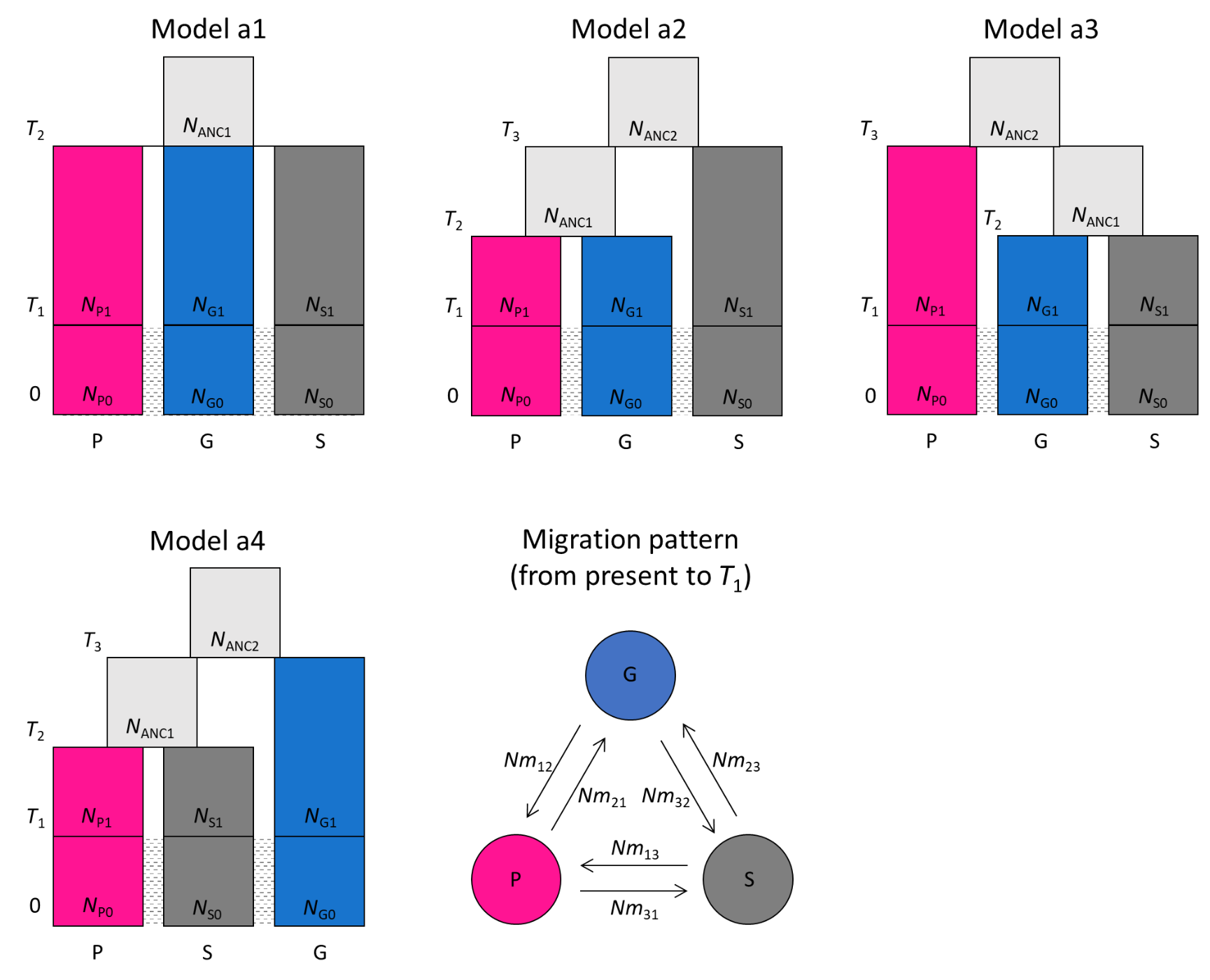
**

**Fig. S3** Details of the compared population demographic models in Fig. S2a. *N*, effective population size; *T*, event time; *Nm*_ij_, number of migrants per generation from species j to i (its direction is forward-in-time). The period shown in dashed lines assumes migration between species.


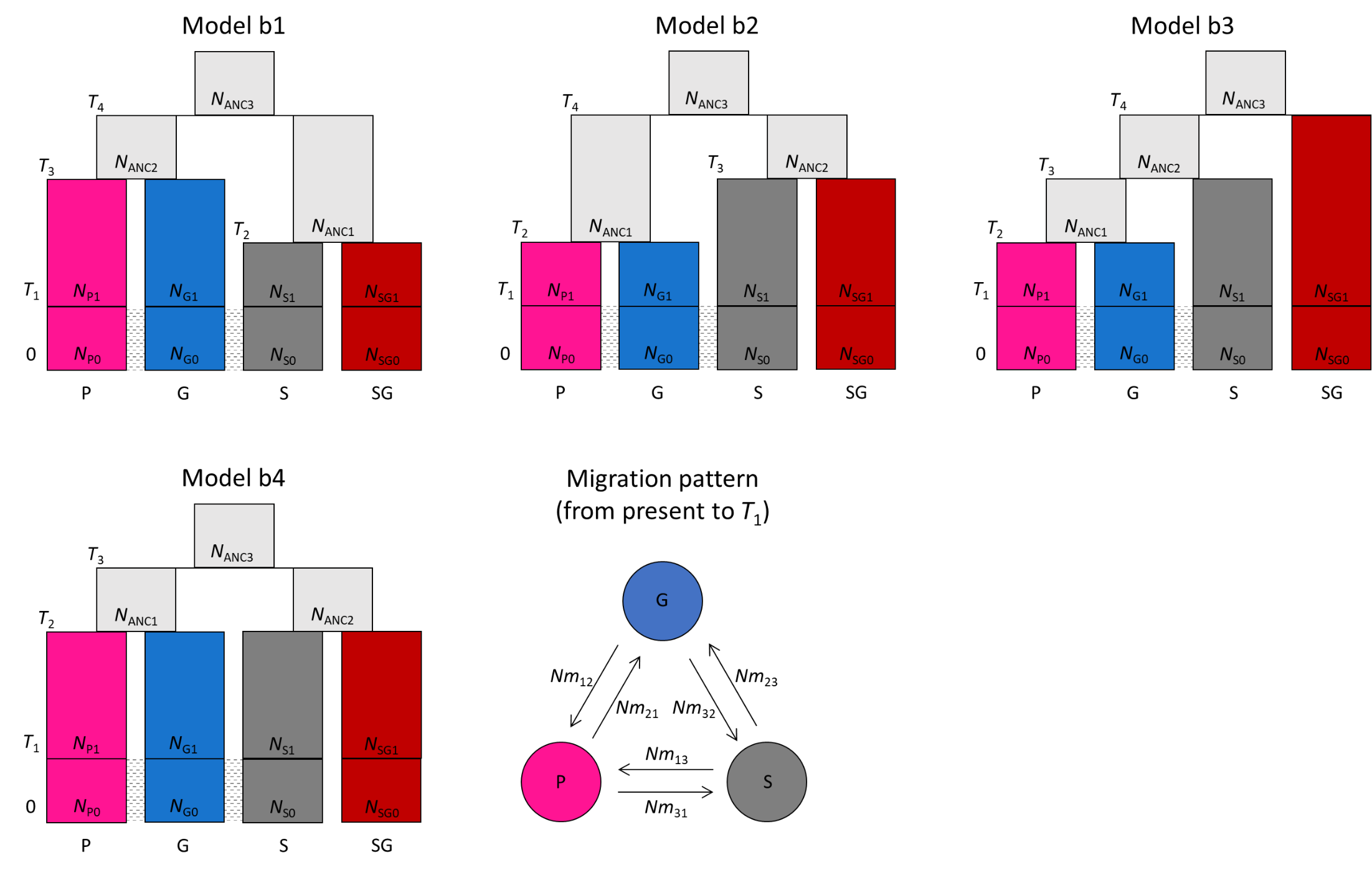


**Figure S4** Details of the compared population demographic models in Fig. S2b. *N*, effective population size; *T*, event time; *Nm*_ij_, number of migrants per generation from species/ecotype j to i (its direction is forward-in-time). The period shown in dashed lines assumes migration between species/ecotypes.


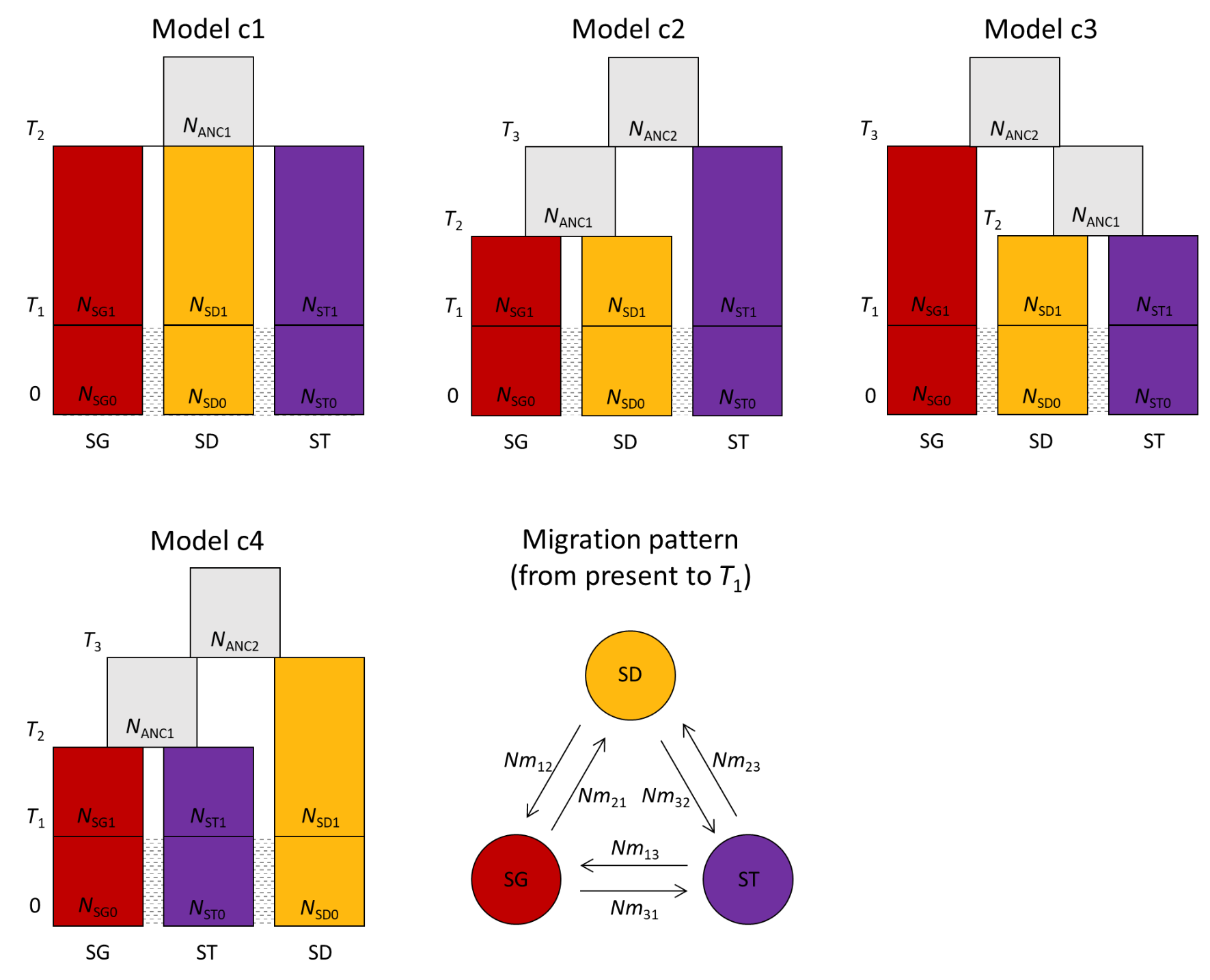


**Fig. S5** Details of the compared population demographic models in Fig. S2c. *N*, effective population size; *T*, event time; *Nm*_ij_, number of migrants per generation from ecotype j to i (its direction is forward-in-time). The period shown in dashed lines assumes migration between ecotypes.


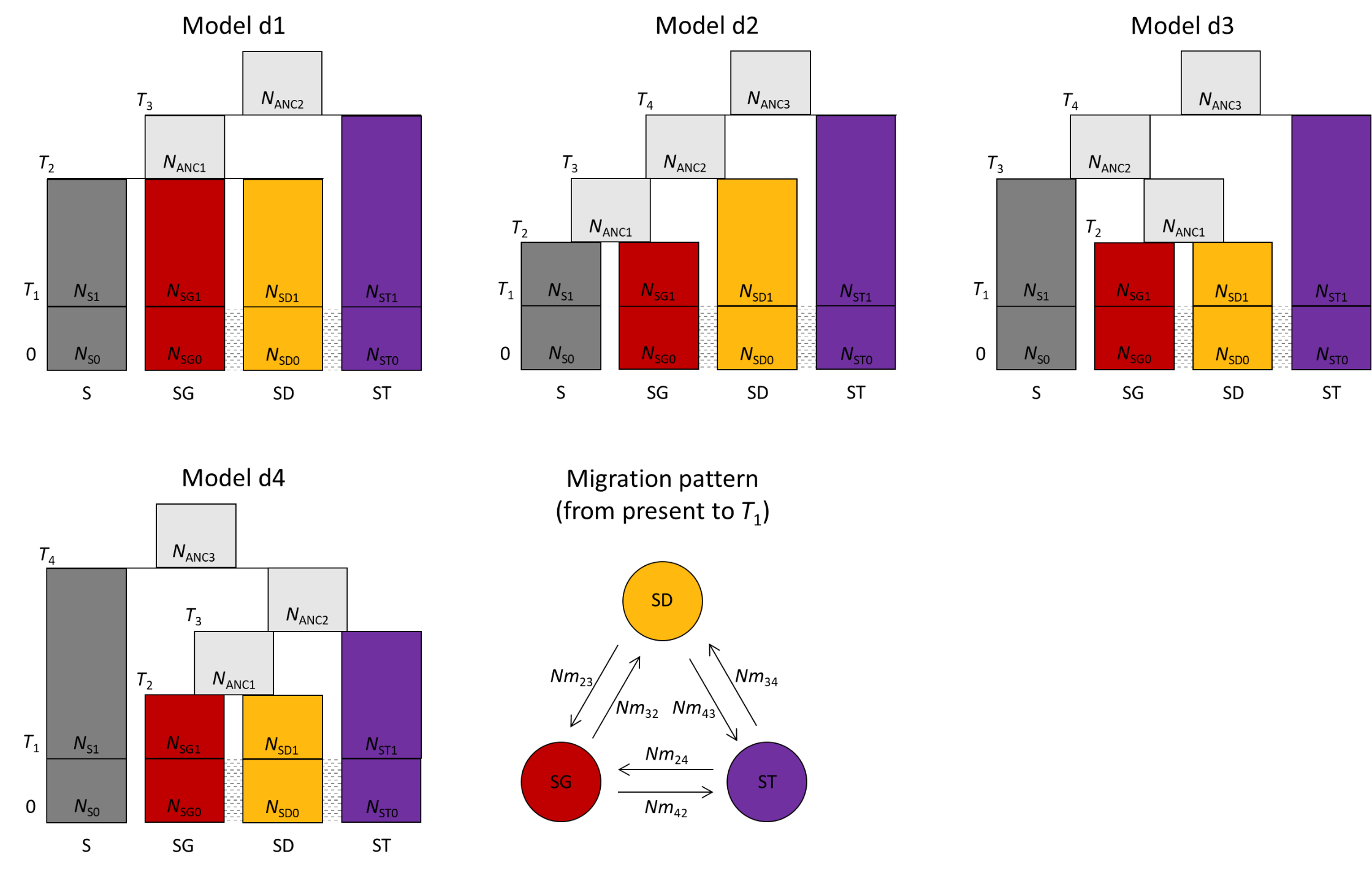


**Fig. S6** Details of the compared population demographic models in Fig. S2d. *N*, effective population size; *T*, event time; *Nm*_ij_, number of migrants per generation from ecotype j to i (its direction is forward-in-time). The period shown in dashed lines assumes migration between ecotypes.


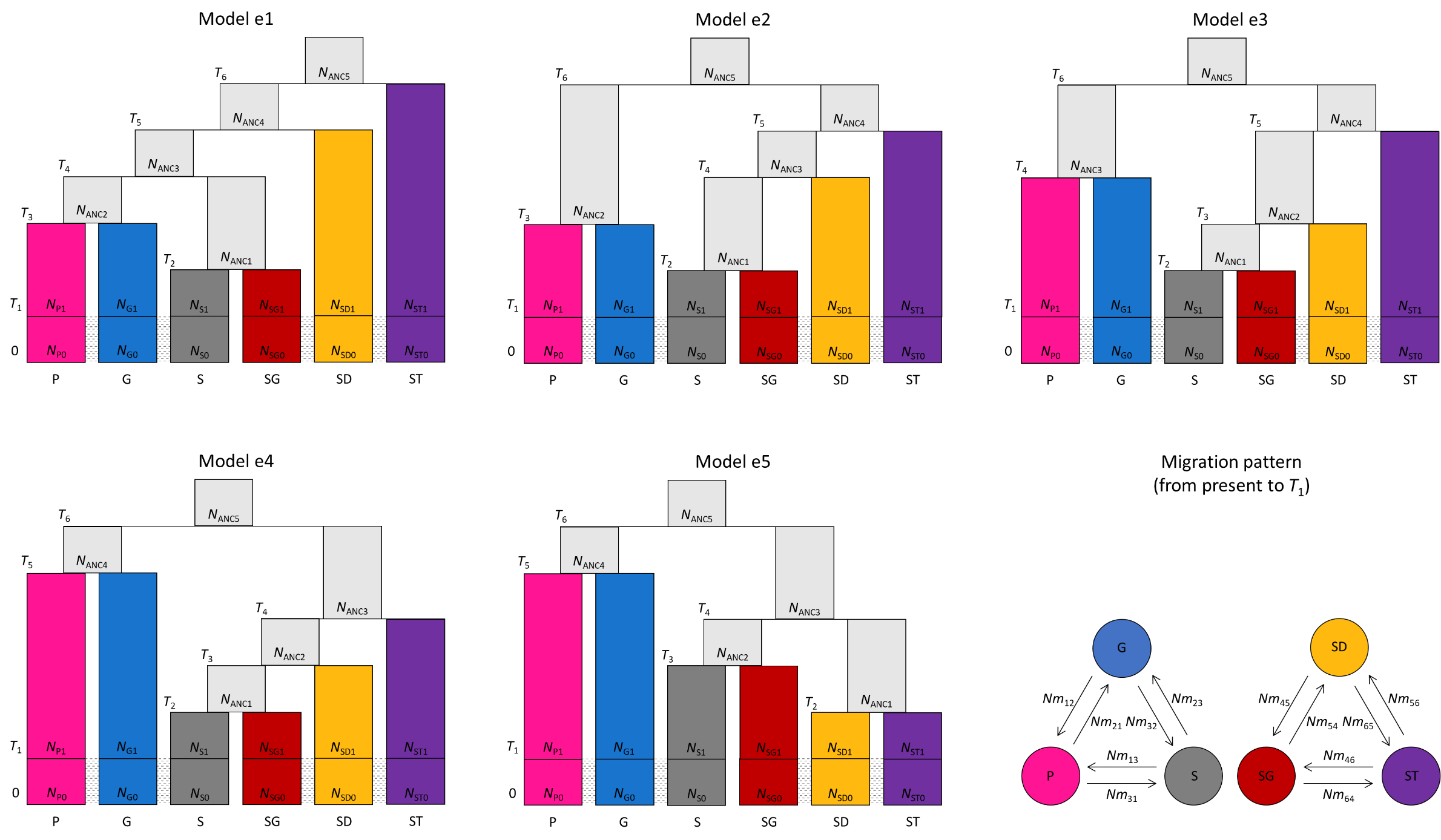


**Fig. S7** Details of the compared population demographic models in Fig. S2e. *N*, effective population size; *T*, event time; *Nm*_ij_, number of migrants per generation from species/ecotype j to i (its direction is forward-in-time). The period shown in dashed lines assumes migration between species/ecotypes.


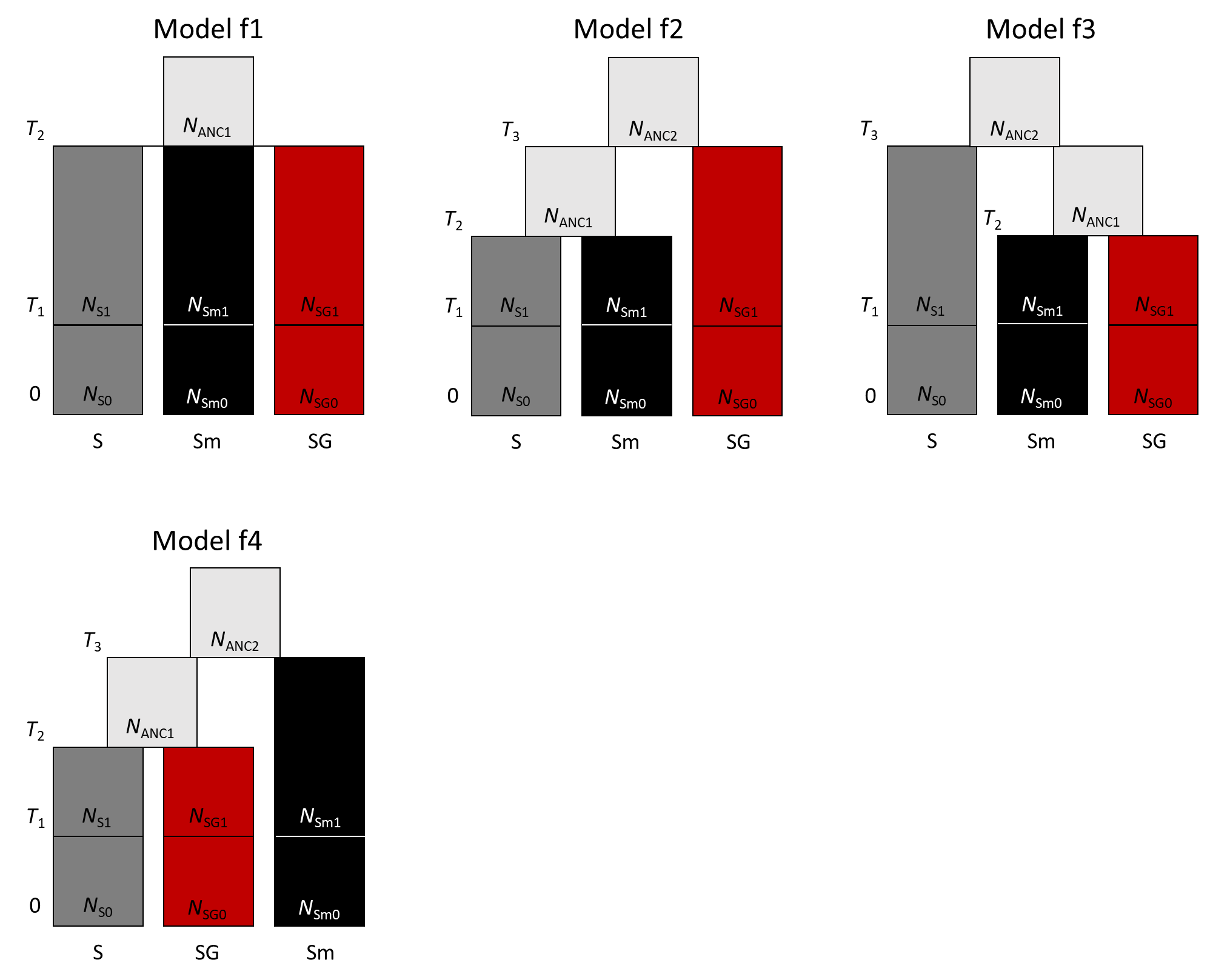


**Fig. S8** Details of the compared population demographic models in Fig. S2f. *N*, effective population size; *T*, event time.


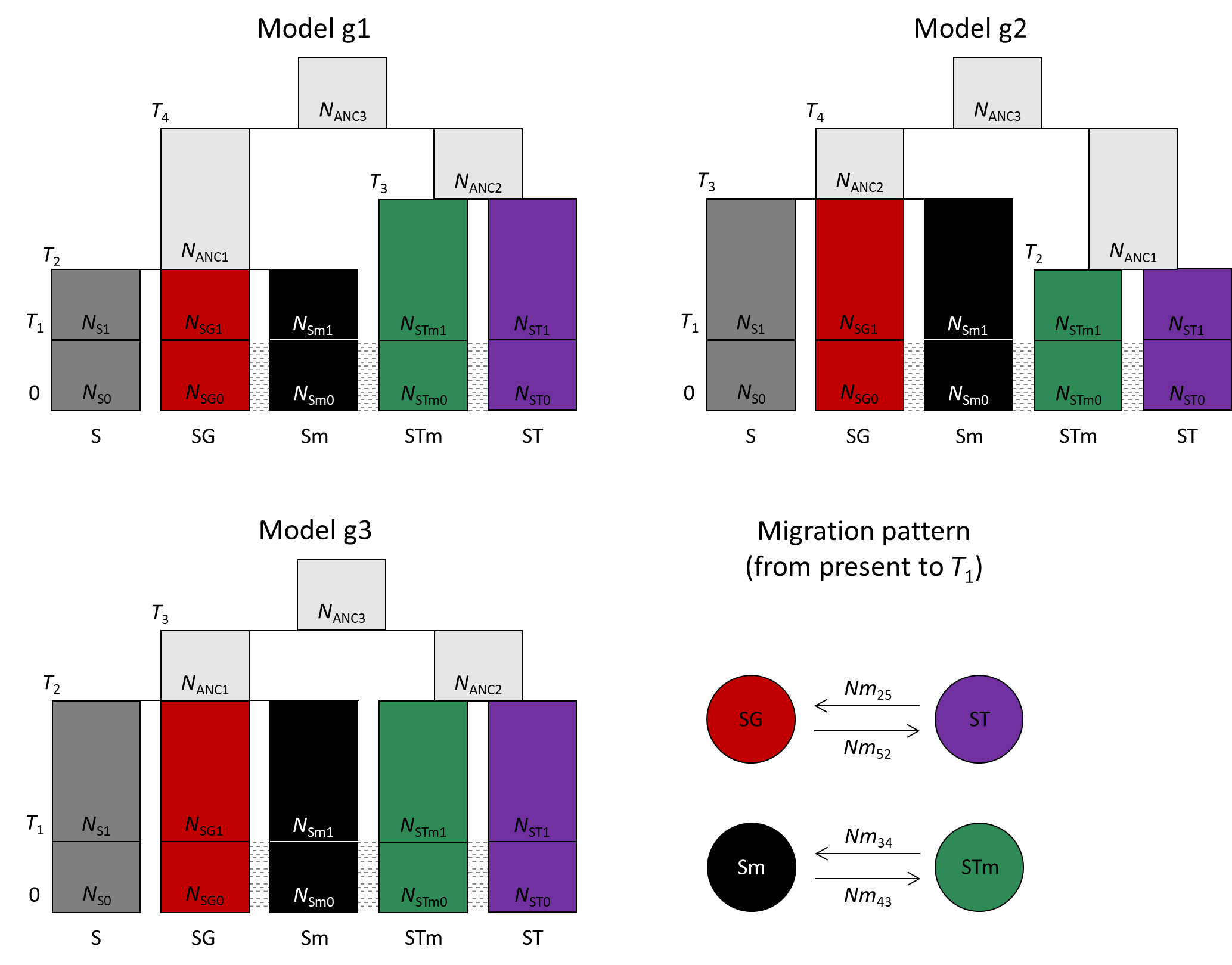


**Fig. S9** Details of the compared population demographic models in Fig. S2g. *N*, effective population size; *T*, event time; *Nm*_ij_, number of migrants per generation from ecotype j to i (its direction is forward-in-time). The period shown in dashed lines assumes migration between ecotypes.


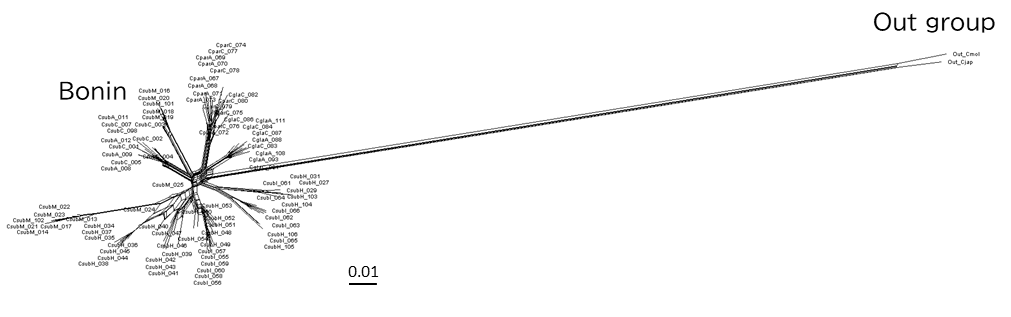


**Fig. S10** Neighbor-net network tree showing the genetic relationship between genus *Callicarpa* populations in the Bonin Islands and the outgroup in mainland Japan.


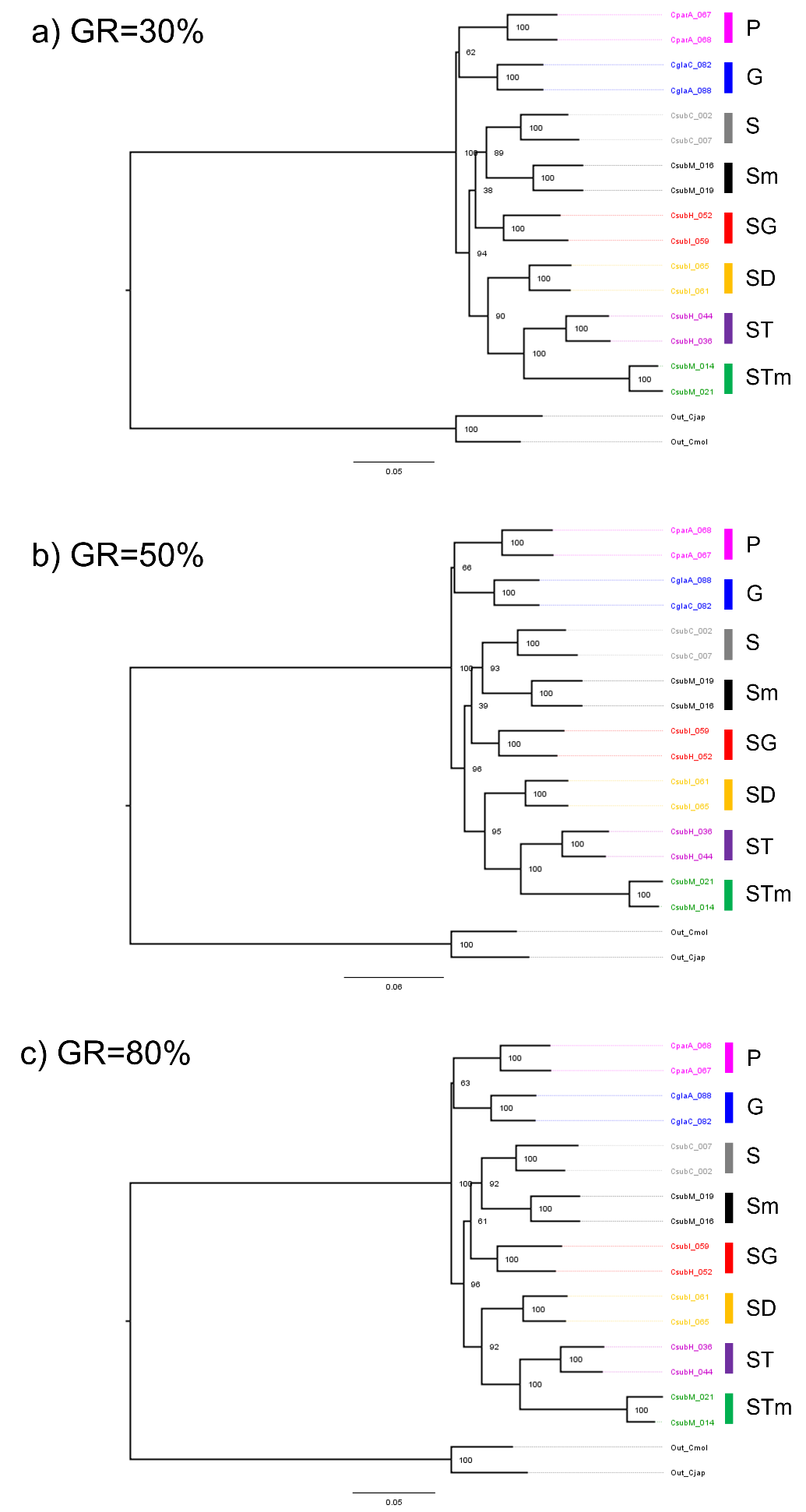


**Fig. S11** Phylogenetic tree of the *Callicarpa* genus in the Bonin Islands using the two individuals with the highest number of SNPs from each genetic group. a), b), c) show trees constructed where SNP genotyping rates (GR) were 30%, 50%, and 80% or more, respectively, including ecotype SH. Phylogenetic clade colors correspond to those used in Figs. 1–3. Out_Cmol and Out_Cjap are outgroups.


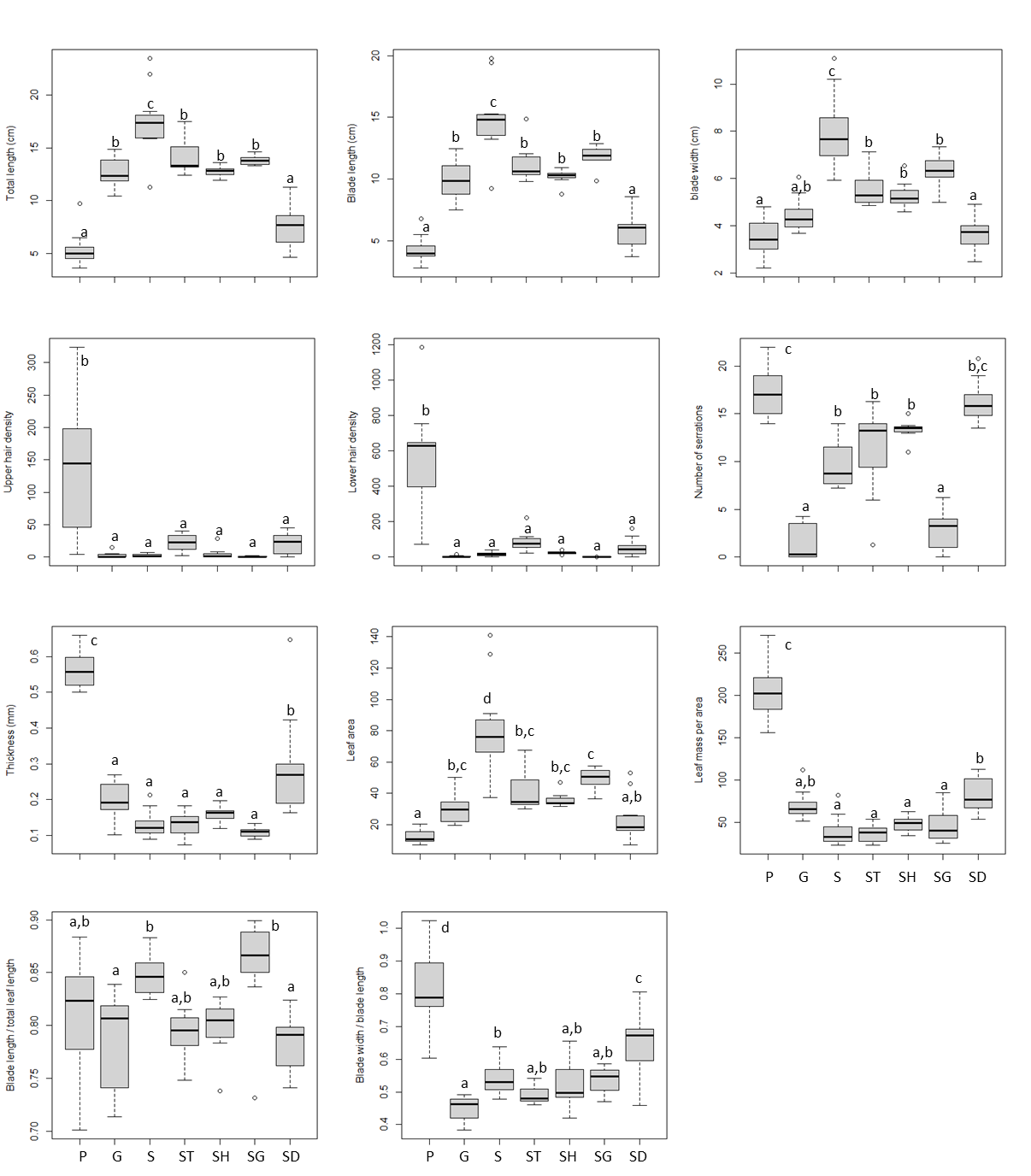


**Fig. S12** Eleven leaf morphological traits of each species/ecotype of genus *Callicarpa* in the Bonin Islands. Different letters indicate significant difference among ecotypes (*p* < 0.05, pairwise t-test with Bonferroni correction).

**Table S1** Summary of conditions in SNP filtering for each dataset and the number of individuals and SNPs used in each analysis.

| Data set name | Reference data | Outgroups | SNP detection | SNPs across all individuals | Missing rate of individuals | Depth | Minor allele frequency | Observed heterozygosity | No. individuals (no. species/ ecotypes) | | Multiple or one SNP /locus | Total sites | No. Loci | No. SNPs used | Analysis |
| --- | --- | --- | --- | --- | --- | --- | --- | --- | --- | --- | --- | --- | --- | --- | --- |
| De novo dataset | not used | not included | dDocent | >80% | >50% | >10 | >0.05 | <0.5 | 89 | (9) | one |  |  | 2336 | ADIMIXTURE |
| Referenced dataset | used | included | dDocent | >80% | >50% | >10 | >0 | <0.5 | 91 | (9) | multiple |  |  | 3566 | SplitsTree with SH |
|  |  |  |  |  |  |  |  |  | 84 | (8) | multiple |  |  | 3498 | SplitsTree without SH |
|  |  |  |  | >30% | >50% | >10 | >0 | <0.5 | 18 | (8) | multiple |  |  | 2953 | RAxML-ng without SH |
|  |  |  |  | >50% | >50% | >10 | >0 | <0.5 | 18 | (8) | multiple |  |  | 2861 | RAxML-ng without SH |
|  |  |  |  | >80% | >50% | >10 | >0 | <0.5 | 18 | (8) | multiple |  |  | 2723 | RAxML-ng without SH |
| Demography dataset | used | included | dDocent, Stacks | — | — | >0 | <0.5 | <0.5 | 35 | (3) | multiple | 794103 | 14996 | 11629 | fastsimcoal2, model a |
|  |  |  |  |  |  |  |  |  | 48 | (4) | multiple | 767491 | 14487 | 14951 | fastsimcoal2, model b |
|  |  |  |  |  |  |  |  |  | 33 | (3) | multiple | 821577 | 15527 | 10796 | fastsimcoal2, model c |
|  |  |  |  |  |  |  |  |  | 44 | (4) | multiple | 805418 | 15218 | 13086 | fastsimcoal2, model d |
|  |  |  |  |  |  |  |  |  | 68 | (6) | multiple | 716748 | 13518 | 17176 | fastsimcoal2, model e |
|  |  |  |  |  |  |  |  |  | 29 | (3) | multiple | 926850 | 17551 | 11689 | fastsimcoal2, model f |
|  |  |  |  |  |  |  |  |  | 43 | (5) | multiple | 816571 | 15436 | 13204 | fastsimcoal2, model g |

**Table S2** Number of parameters (NP), log-likelihood (LL; in log_10_ scale), Akaike’s information criterion (AIC) and maximum likelihood estimates of parameters for three-species divergence models (three Chichijima species; Fig. S2a). The best model was shown in bold.

| Parameter | Model a1 | **Model a2** | Model a3 | Model a4 |
| --- | --- | --- | --- | --- |
| NP | 15 | **17** | 17 | 17 |
| LL | -98190 | **-98163** | -98192 | -98189 |
| AIC | 452214 | **452090** | 452223 | 452213 |
| *N*_P0_ | 32055 | **33460** | 32939 | 32138 |
| *N*_G0_ | 59804 | **56508** | 53759 | 54887 |
| *N*_S0_ | 22916 | **19809** | 22351 | 23874 |
| *N*_P1_ | 11327 | **5851** | 10744 | 10187 |
| *N*_G1_ | 10404 | **5058** | 8406 | 9849 |
| *N*_S1_ | 29973 | **153661** | 29662 | 25561 |
| *N*_ANC1_ | 12615 | **14915** | 193964 | 81337 |
| *N*_ANC2_ | — | **12205** | 13130 | 11760 |
| *T*_1_ (kya) | 71.3 | **82.6** | 82.5 | 83.5 |
| *T*_2_ (kya) | 146.6 | **112.9** | 151.3 | 154.9 |
| *T*_3_ (kya) | — | **171.2** | 151.8 | 156.3 |
| *Nm*_12_ | 0.265 | **0.152** | 0.248 | 0.253 |
| *Nm*_13_ | 0.215 | **0.262** | 0.177 | 0.198 |
| *Nm*_21_ | 0.268 | **0.166** | 0.223 | 0.220 |
| *Nm*_23_ | 0.199 | **0.253** | 0.206 | 0.183 |
| *Nm*_31_ | 0.081 | **0.082** | 0.073 | 0.086 |
| *Nm*_32_ | 0.101 | **0.109** | 0.109 | 0.097 |

*N*, effective population size; *T*, event time; *Nm*_ij_, number of migrants per generation from species j to i (its direction is forward-in-time). Numbers in the subscript of *Nm* are 1, P; 2, G; 3, S.

**Table S3** Number of parameters (NP), log-likelihood (LL; in log_10_ scale), Akaike’s information criterion (AIC) and maximum likelihood estimates of parameters for four-species/ecotype divergence models (three Chichijima and one Hahajima species/ecotypes; Fig. S2b). The best model was shown in bold.

| Parameter | **Model b1** | Model b2 | Model b3 | Model b4 |
| --- | --- | --- | --- | --- |
| NP | **21** | 21 | 21 | 20 |
| LL | **-199464** | -199537 | -199640 | -199530 |
| AIC | **918608** | 918945 | 919417 | 918911 |
| *N*_P0_ | **35680** | 37099 | 33698 | 36565 |
| *N*_G0_ | **66715** | 62365 | 59028 | 73099 |
| *N*_S0_ | **20103** | 21513 | 22947 | 21824 |
| *N*_SG0_ | **70258** | 60096 | 61502 | 68406 |
| *N*_P1_ | **15523** | 1839 | 7486 | 1323 |
| *N*_G1_ | **15993** | 1196 | 5373 | 1121 |
| *N*_S1_ | **30816** | 106963 | 198085 | 282347 |
| *N*_SG1_ | **2747** | 64984 | 47938 | 9286 |
| *N*_ANC1_ | **57992** | 27571 | 1645 | 31503 |
| *N*_ANC2_ | **2393** | 43603 | 130923 | 50630 |
| *N*_ANC3_ | **13965** | 20013 | 16625 | 12752 |
| *T*_1_ (kya) | **54.5** | 69.0 | 72.8 | 66.2 |
| *T*_2_ (kya) | **56.2** | 74.4 | 105.1 | 71.1 |
| *T*_3_ (kya) | **129.9** | 74.9 | 108.0 | 147.0 |
| *T*_4_ (kya) | **135.8** | 150.1 | 108.6 | — |
| *Nm*_12_ | **0.155** | 0.079 | 0.156 | 0.064 |
| *Nm*_13_ | **0.363** | 0.308 | 0.245 | 0.379 |
| *Nm*_21_ | **0.114** | 0.075 | 0.114 | 0.073 |
| *Nm*_23_ | **0.485** | 0.475 | 0.356 | 0.597 |
| *Nm*_31_ | **0.052** | 0.044 | 0.019 | 0.037 |
| *Nm*_32_ | **0.040** | 0.048 | 0.028 | 0.028 |

*N*, effective population size; *T*, event time; *Nm*_ij_, number of migrants per generation from species/ecotype j to i (its direction is forward-in-time). Numbers in the subscript of *Nm* are 1, P; 2, G; 3, S.

**Table S4** Number of parameters (NP), log-likelihood (LL; in log_10_ scale), Akaike’s information criterion (AIC) and maximum likelihood estimates of parameters for three-ecotype divergence models (three Hahajima ecotypes; Fig. S2c). The best model was shown in bold.

| Parameter | Model c1 | **Model c2** | Model c3 | Model c4 |
| --- | --- | --- | --- | --- |
| NP | 15 | **17** | 17 | 17 |
| LL | -95690 | **-95674** | -95689 | -95689 |
| AIC | 440697 | **440628** | 440699 | 440696 |
| *N*_SG0_ | 51515 | **56903** | 53170 | 50680 |
| *N*_SD0_ | 14172 | **15660** | 15118 | 14327 |
| *N*_ST0_ | 17240 | **17689** | 16456 | 16998 |
| *N*_SG1_ | 12200 | **16711** | 11270 | 1859 |
| *N*_SD1_ | 22617 | **27277** | 18536 | 44806 |
| *N*_ST1_ | 9186 | **12079** | 9766 | 1657 |
| *N*_ANC1_ | 10110 | **2878** | 317562 | 286983 |
| *N*_ANC2_ | — | **12468** | 10160 | 8411 |
| *T*_1_ (kya) | 100.9 | **79.3** | 98.2 | 116.8 |
| *T*_2_ (kya) | 193.7 | **166.9** | 193.4 | 131.3 |
| *T*_3_ (kya) | — | **178.0** | 194.5 | 224.5 |
| *Nm*_12_ | 0.289 | **0.286** | 0.277 | 0.283 |
| *Nm*_13_ | 0.202 | **0.277** | 0.194 | 0.195 |
| *Nm*_21_ | 0.243 | **0.181** | 0.251 | 0.230 |
| *Nm*_23_ | 0.291 | **0.333** | 0.318 | 0.269 |
| *Nm*_31_ | 0.075 | **0.053** | 0.069 | 0.069 |
| *Nm*_32_ | 0.046 | **0.048** | 0.052 | 0.056 |

*N*, effective population size; *T*, event time; *Nm*_ij_, number of migrants per generation from ecotype j to i (its direction is forward-in-time). Numbers in the subscript of *Nm* are 1, SG; 2, SD; 3, ST.

**Table S5** Number of parameters (NP), log-likelihood (LL; in log_10_ scale), Akaike’s information criterion (AIC) and maximum likelihood estimates of parameters for four-ecotype divergence models (one Chichijima and three Hahajima ecotypes; Fig. S2d). The best model was shown in bold.

| Parameter | Model d1 | **Model d2** | Model d3 | Model d4 |
| --- | --- | --- | --- | --- |
| NP | 19 | **21** | 21 | 21 |
| LL | -189086 | **-189070** | -189088 | -189177 |
| AIC | 870812 | **870740** | 870825 | 871234 |
| *N*_S0_ | 23317 | **23935** | 24391 | 17130 |
| *N*_SG0_ | 57664 | **59395** | 58141 | 65900 |
| *N*_SD0_ | 15416 | **15413** | 16217 | 15932 |
| *N*_ST0_ | 16464 | **16641** | 15790 | 17434 |
| *N*_S1_ | 558581 | **109363** | 368378 | 446180 |
| *N*_SG1_ | 1353 | **1465** | 1457 | 21173 |
| *N*_SD1_ | 6633 | **3134** | 6007 | 28372 |
| *N*_ST1_ | 10928 | **11505** | 10703 | 8952 |
| *N*_ANC1_ | 51792 | **4178** | 629642 | 12849 |
| *N*_ANC2_ | 12482 | **78873** | 53288 | 492345 |
| *N*_ANC3_ | — | **12640** | 12584 | 15999 |
| *T*_1_ (kya) | 65.4 | **64.3** | 67.5 | 42.4 |
| *T*_2_ (kya) | 69.1 | **66.8** | 72.1 | 121.4 |
| *T*_3_ (kya) | 180.1 | **68.8** | 72.1 | 121.8 |
| *T*_4_ (kya) | — | **171.4** | 173.7 | 122.9 |
| *Nm*_23_ | 0.451 | **0.260** | 0.638 | 0.622 |
| *Nm*_24_ | 0.177 | **0.222** | 0.113 | 0.287 |
| *Nm*_32_ | 0.088 | **0.136** | 0.092 | 0.116 |
| *Nm*_34_ | 0.318 | **0.301** | 0.281 | 0.329 |
| *Nm*_42_ | 0.071 | **0.085** | 0.072 | 0.053 |
| *Nm*_43_ | 0.083 | **0.071** | 0.086 | 0.097 |

*N*, effective population size; *T*, event time; *Nm*_ij_, number of migrants per generation from ecotype j to i (its direction is forward-in-time). Numbers in the subscript of *Nm* are 2, SG; 3, SD; 4, ST.

**Table S6** Number of parameters (NP), log-likelihood (LL; in log_10_ scale), Akaike’s information criterion (AIC) and maximum likelihood estimates of parameters for six-species/ecotype divergence models (three Chichijima and three Hahajima species/ecotypes; Fig. S2e). The best model was shown in bold.

| Parameter | **Model e1** | Model e2 | Model e3 | Model e4 | Model e5 |
| --- | --- | --- | --- | --- | --- |
| NP | **35** | 35 | 35 | 35 | 35 |
| LL | **-437603** | -437651 | -437652 | -437646 | -437674 |
| AIC | **2015305** | 2015526 | 2015533 | 2015506 | 2015635 |
| *N*_P0_ | **37058** | 33186 | 36172 | 33542 | 33919 |
| *N*_G0_ | **68868** | 61797 | 64239 | 65238 | 63686 |
| *N*_S0_ | **23146** | 22424 | 21566 | 21606 | 22206 |
| *N*_SG0_ | **57136** | 61152 | 61155 | 60752 | 54981 |
| *N*_SD0_ | **15777** | 15487 | 12824 | 14266 | 15177 |
| *N*_ST0_ | **18372** | 18373 | 19454 | 18662 | 19321 |
| *N*_P1_ | **1286** | 13045 | 17299 | 15989 | 10856 |
| *N*_G1_ | **1113** | 11524 | 14826 | 14478 | 8997 |
| *N*_S1_ | **250728** | 60932 | 22188 | 117858 | 124052 |
| *N*_SG1_ | **1088** | 5223 | 2248 | 8702 | 7764 |
| *N*_SD1_ | **115352** | 400363 | 466393 | 21141 | 63381 |
| *N*_ST1_ | **8983** | 7339 | 7512 | 8360 | 1150 |
| *N*_ANC1_ | **3442** | 22249 | 32586 | 5415 | 588831 |
| *N*_ANC2_ | **84823** | 2295 | 46919 | 285856 | 22503 |
| *N*_ANC3_ | **155876** | 206092 | 1091 | 389589 | 77772 |
| *N*_ANC4_ | **68502** | 142787 | 100068 | 3254 | 165065 |
| *N*_ANC5_ | **13095** | 15569 | 15185 | 15031 | 15620 |
| *T*_1_ (kya) | **70.2** | 67.7 | 53.8 | 57.5 | 68.7 |
| *T*_2_ (kya) | **72.9** | 73.6 | 56.2 | 72.9 | 77.9 |
| *T*_3_ (kya) | **76.0** | 132.2 | 133.6 | 80.5 | 80.4 |
| *T*_4_ (kya) | **76.2** | 132.4 | 134.7 | 132.6 | 130.2 |
| *T*_5_ (kya) | **76.9** | 134.3 | 135.6 | 133.4 | 131.2 |
| *T*_6_ (kya) | **170.7** | 134.6 | 136.6 | 138.5 | 131.3 |
| *Nm*_12_ | **0.210** | 0.212 | 0.110 | 0.117 | 0.196 |
| *Nm*_13_ | **0.264** | 0.260 | 0.348 | 0.334 | 0.299 |
| *Nm*_21_ | **0.126** | 0.104 | 0.211 | 0.073 | 0.131 |
| *Nm*_23_ | **0.424** | 0.427 | 0.521 | 0.560 | 0.455 |
| *Nm*_31_ | **0.026** | 0.067 | 0.092 | 0.021 | 0.079 |
| *Nm*_32_ | **0.021** | 0.084 | 0.039 | 0.056 | 0.060 |
| *Nm*_45_ | **0.511** | 0.434 | 0.632 | 0.557 | 0.393 |
| *Nm*_46_ | **0.152** | 0.150 | 0.113 | 0.166 | 0.122 |
| *Nm*_54_ | **0.132** | 0.176 | 0.196 | 0.230 | 0.177 |
| *Nm*_56_ | **0.317** | 0.306 | 0.307 | 0.286 | 0.282 |
| *Nm*_64_ | **0.154** | 0.089 | 0.126 | 0.085 | 0.088 |
| *Nm*_65_ | **0.020** | 0.069 | 0.038 | 0.052 | 0.040 |

*N*, effective population size; *T*, event time; *Nm*_ij_, number of migrants per generation from species/ecotype j to i (its direction is forward-in-time). Numbers in the subscript of *Nm* are 1, P; 2, G; 3, S; 4, SG; 5, SD; 6, ST.

**Table S7** Maximum likelihood estimate (MLE) and 95% confidence interval (CI) of parameters in the best six-species/ecotype divergence model (three Chichijima and three Hahajima species/ecotypes; Model e1 in Fig. S2e).

| Parameter | MLE (95% CI) |
| --- | --- |
| *N*_P0_ | 37058 (33321–41169) |
| *N*_G0_ | 68868 (61608–79279) |
| *N*_S0_ | 23146 (20933–25593) |
| *N*_SG0_ | 57136 (51208–65228) |
| *N*_SD0_ | 15777 (15558–17378) |
| *N*_ST0_ | 18372 (16630–21228) |
| *N*_P1_ | 1286 (1115–1682) |
| *N*_G1_ | 1113 (1103–1469) |
| *N*_S1_ | 250728 (8378–438027) |
| *N*_SG1_ | 1088 (1086–1447) |
| *N*_SD1_ | 115352 (9628–504990) |
| *N*_ST1_ | 8983 (7315–10697) |
| *N*_ANC1_ | 3442 (2696–10152) |
| *N*_ANC2_ | 84823 (2944–376536) |
| *N*_ANC3_ | 155876 (7236–317037) |
| *N*_ANC4_ | 68502 (58145–91533) |
| *N*_ANC5_ | 13095 (11911–14397) |
| *T*_1_ (kya)^*^ | 70.2 |
| *T*_2_ (kya) | 72.9 (72.5–73.8) |
| *T*_3_ (kya) | 76.0 (75.3–77.4) |
| *T*_4_ (kya) | 76.2 (76.0–81.4) |
| *T*_5_ (kya) | 76.9 (76.6–83.1) |
| *T*_6_ (kya) | 170.7 (162.2–183.4) |
| *Nm*_12_ | 0.210 (0.140–0.252) |
| *Nm*_13_ | 0.264 (0.198–0.341) |
| *Nm*_21_ | 0.126 (0.049–0.182) |
| *Nm*_23_ | 0.424 (0.343–0.530) |
| *Nm*_31_ | 0.026 (0.014–0.059) |
| *Nm*_32_ | 0.021 (0.016–0.058) |
| *Nm*_45_ | 0.511 (0.411–0.656) |
| *Nm*_46_ | 0.152 (0.075–0.206) |
| *Nm*_54_ | 0.132 (0.076–0.185) |
| *Nm*_56_ | 0.317 (0.259–0.375) |
| *Nm*_64_ | 0.154 (0.096–0.186) |
| *Nm*_65_ | 0.020 (0.014–0.070) |

^*^ As only this parameter was not updated in the parametric bootstrapping, 95% CI was not be able to be estimated.

*N*, effective population size. Its unit is a number of diploid individuals; *T*, event time; *Nm*_ij_, number of migrants per generation from j to i. Direction of migration is the movement of individuals (i.e., forward-in-time). Numbers in the subscript of *Nm* are 1, P; 2, G; 3, S; 4, SG; 5, SD; 6, ST.

**Table S8** Maximum likelihood estimate (MLE) and 95% confidence interval (CI) of relative population size (*N*_0_/*N*_1_) in the best models e1 and g2. Species/ecotypes that significantly changed its population size recently (i.e., ecotypes do not contain 1 in its CI) are shown in bold.

| Model | Species/Ecotype | MLE (95%CI) |
| --- | --- | --- |
| Model e1 | **P** | **28.82 (21.53–33.83)** |
|  | **G** | **61.88 (46.35–69.95)** |
|  | S | 0.09 (0.05–2.97) |
|  | **SG** | **52.51 (38.34–58.59)** |
|  | SD | 0.14 (0.03–1.81) |
|  | **ST** | **2.05 (1.68–2.59)** |
| Model g2 | **S** | **0.16 (0.05–0.34)** |
|  | **SG** | **2.33 (1.86–3.20)** |
|  | **Sm** | **0.03 (0.01–0.16)** |
|  | **STm** | **0.02 (0.01–0.26)** |
|  | **ST** | **1.82 (1.19–2.53)** |

**Table S9** Number of parameters (NP), log-likelihood (LL; in log_10_ scale), Akaike’s information criterion (AIC) and maximum likelihood estimates of parameters for three-ecotype divergence models (one Chichijima, one Hahajima and one Mukojima ecotypes; Fig. S2f). The best model was shown in bold.

| Parameter | **Model f1** | Model f2 | Model f3 | Model f4 |
| --- | --- | --- | --- | --- |
| NP | **9** | 11 | 11 | 11 |
| LL | **-101076** | -101077 | -101079 | -101079 |
| AIC | **465491** | 465501 | 465508 | 465508 |
| *N*_S0_ | **6333** | 5341 | 6850 | 6681 |
| *N*_Sm0_ | **1232** | 1147 | 1197 | 1103 |
| *N*_SG0_ | **72057** | 225869 | 191407 | 168898 |
| *N*_S1_ | **39109** | 41453 | 37966 | 43141 |
| *N*_Sm1_ | **49201** | 40036 | 49222 | 120582 |
| *N*_SG1_ | **53413** | 52708 | 50634 | 48622 |
| *N*_ANC1_ | **16069** | 18018 | 114821 | 3178 |
| *N*_ANC2_ | **—** | 16593 | 16080 | 16643 |
| *T*_1_ (kya) | **3.1** | 2.6 | 3.3 | 3.5 |
| *T*_2_ (kya) | **99.6** | 98.3 | 97.3 | 100.0 |
| *T*_3_ (kya) | **—** | 99.2 | 99.1 | 101.0 |

*N*, effective population size; *T*, event time.

**Table S10** Number of parameters (NP), log-likelihood (LL; in log_10_ scale), Akaike’s information criterion (AIC) and maximum likelihood estimates of parameters for five-ecotype divergence models (one Chichijima, two Hahajima and two Mukojima ecotypes; Fig. S2g). The best model was shown in bold.

| Parameter | Model g1 | **Model g2** | Model g3 |
| --- | --- | --- | --- |
| NP | 21 | **21** | 20 |
| LL | -266581 | **-266580** | -266583 |
| AIC | 1227693 | **1227687** | 1227701 |
| *N*_S0_ | 13158 | **18954** | 13276 |
| *N*_SG0_ | 76187 | **63698** | 77553 |
| *N*_Sm0_ | 3602 | **6596** | 3183 |
| *N*_STm0_ | 4160 | **4410** | 3112 |
| *N*_ST0_ | 17792 | **19978** | 15442 |
| *N*_S1_ | 73731 | **120878** | 55620 |
| *N*_SG1_ | 35559 | **27386** | 39983 |
| *N*_Sm1_ | 228904 | **210093** | 103828 |
| *N*_STm1_ | 13422 | **187761** | 15599 |
| *N*_ST1_ | 16268 | **10982** | 17635 |
| *N*_ANC1_ | 21590 | **17710** | 24506 |
| *N*_ANC2_ | 13267 | **27867** | 13214 |
| *N*_ANC3_ | 14940 | **14454** | 15119 |
| *T*_1_ (kya) | 16.8 | **37.1** | 14.3 |
| *T*_2_ (kya) | 90.9 | **81.4** | 89.4 |
| *T*_3_ (kya) | 94.6 | **82.3** | 154.9 |
| *T*_4_ (kya) | 155.9 | **168.8** | — |
| *Nm*_25_ | 0.903 | **0.387** | 0.785 |
| *Nm*_34_ | 0.101 | **0.102** | 0.106 |
| *Nm*_43_ | 0.091 | **0.066** | 0.092 |
| *Nm*_52_ | 0.171 | **0.129** | 0.200 |

*N*, effective population size; *T*, event time; *Nm*_ij_, number of migrants per generation from ecotype j to i (its direction is forward-in-time). Numbers in the subscript of *Nm* are 2, SG; 3, Sm; 4, STm; 5, ST.

**Table S11** Maximum likelihood estimate (MLE) and 95% confidence interval (CI) of parameters in the best five-ecotype divergence model (one Chichijima, two Hahajima and two Mukojima ecotypes; Model g2 in Fig. S2g).

| Parameter | MLE (95% CI) |
| --- | --- |
| *N*_S0_ | 18954 (17167–20916) |
| *N*_SG0_ | 63698 (56965–71285) |
| *N*_Sm0_ | 6596 (6035–7871) |
| *N*_STm0_ | 4410 (4022–5214) |
| *N*_ST0_ | 19978 (16672–22376) |
| *N*_S1_ | 120878 (63942–318195) |
| *N*_SG1_ | 27386 (22347–33613) |
| *N*_Sm1_ | 210093 (47537–490988) |
| *N*_STm1_ | 187761 (19699–437975) |
| *N*_ST1_ | 10982 (8632–14812) |
| *N*_ANC1_ | 17710 (14330–23104) |
| *N*_ANC2_ | 27867 (23183–34450) |
| *N*_ANC3_ | 14454 (13134–15907) |
| *T*_1_ (kya) | 37.1 (33.9–40.5) |
| *T*_2_ (kya) | 81.4 (72.7–86.4) |
| *T*_3_ (kya) | 82.3 (75.0–88.2) |
| *T*_4_ (kya) | 168.8 (160.6–179.1) |
| *Nm*_25_ | 0.387 (0.314–0.463) |
| *Nm*_34_ | 0.102 (0.075–0.120) |
| *Nm*_43_ | 0.066 (0.054–0.081) |
| *Nm*_52_ | 0.129 (0.098–0.158) |

*N*, effective population size. Its unit is a number of diploid individuals; *T*, event time; *Nm*_ij_, number of migrants per generation from j to i. Direction of migration is the movement of individuals (i.e., forward-in-time). Numbers in the subscript of *Nm* are 2, SG; 3, Sm; 4, STm; 5, ST.
